# Synthesis of β-Pyridyl α-Amino Acids: Conformationally Sensitive Charge Transfer-Based Fluorophores

**DOI:** 10.1021/acs.orglett.4c01951

**Published:** 2024-06-12

**Authors:** Leanne M. Riley, Olivia Marshall, Alexander H. Harkiss, Hans M. Senn, Andrew Sutherland

**Affiliations:** School of Chemistry, The Joseph Black Building, University of Glasgow, Glasgow G12 8QQ, United Kingdom

## Abstract

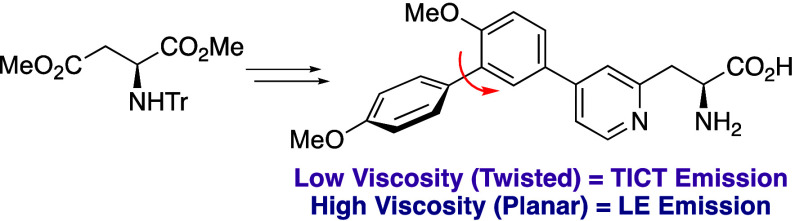

Unnatural α-amino acids with charge transfer-based poly aromatic side chains have been designed as conformationally sensitive fluorophores. These were prepared using a hetero-Diels–Alder reaction and a Knoevenagel–Stobbe process to generate a biaryl pyridyl unit, followed by iron-catalyzed bromination and a Suzuki–Miyaura cross-coupling reaction to complete the triaryl system. A photophysical study led to the discovery of a *p*-methoxy analogue which exhibited viscosity-sensitive fluorescence in which emission could be controlled between twisted and planar conformations.

For the study of biological processes, fluorescence spectroscopy has become a powerful tool for the real-time imaging of molecular events.^1^ One approach to biomedical fluorescent imaging is the conjugation of proteins and peptides with commercially available fluorophores.^2^ However, to minimize disruption of protein structure and function, these large chromophores are typically attached through a linker at the protein terminus, which limits applications. In recent years, this limitation has been overcome using unnatural fluorescent α-amino acids.^3^ Through chemical synthesis, the side chain fluorophores can be tuned for a particular application and using solid phase peptides synthesis or genetic encoding, the amino acids can be incorporated at specific sites of a protein for localized imaging of a biological event. Examples include 4-biphenyl-l-phenylalanine (**1**),^4^ which was incorporated into dihydrofolate reductase for FRET measurement of inhibitor binding and benzoselenadiazole substituted amino acid **2**, used to image synaptosomes in mouse brain tissue (Figure 1a).^5^

**Figure 1:**
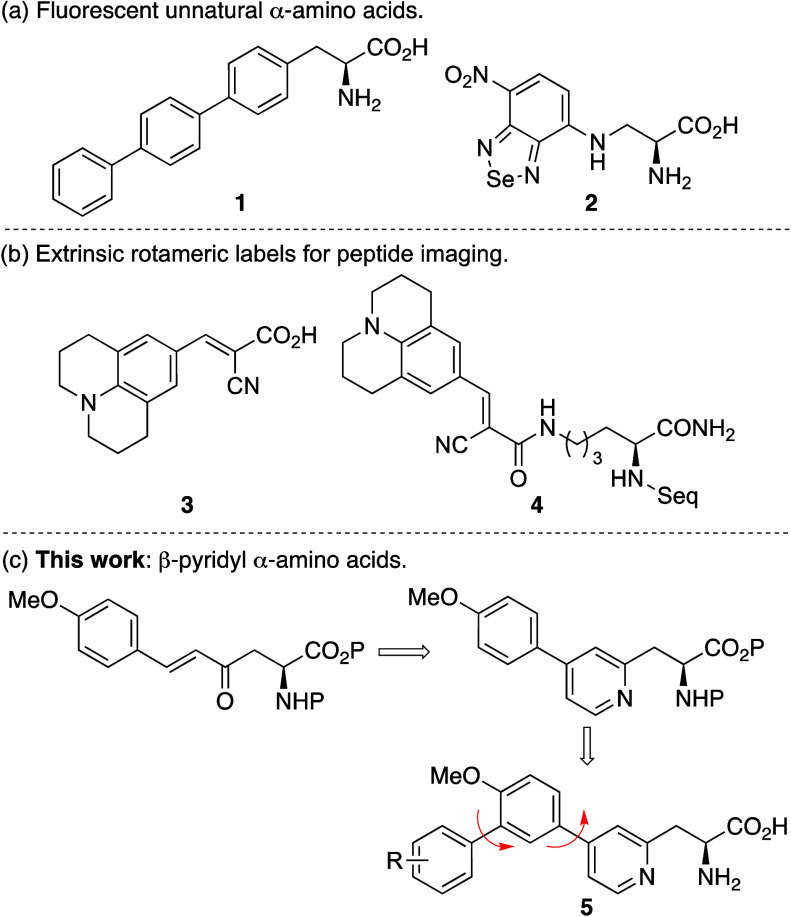
Fluorescent α-amino acids and peptide labels.

Fluorescent molecular rotors that interconvert between planar and twisted conformations can act as environment-sensitive probes under different viscosity conditions.^6^ In biology, as molecular diffusion is dependent on viscosity, fluorescent rotamers have been designed to study lipid membrane, mitochondria and cell function.^6^ Although molecular rotors have been reported for biological investigations, α-amino acids that can act as rotationally controlled viscosity probes and embedded into peptides and proteins for the analysis of specific interactions are rare. Studies do exist in which an extrinsic fluorescent molecular rotor has been attached to a peptide via a chemical spacer or the side chain of an amino acid.^7^ These include julolidine-based molecular rotors such as 9-(2-carboxy-2-cyanovinyl)julolidine **3**,^8^ that on incorporation into a peptide to form fluorescent probes such as **4**, have been used to investigate protein–protein interactions (Figure 1b).^9^ We have reported various novel classes of unnatural α-amino acids that can act as fluorescent probes for chemical biology applications,^10^ including benzotriazinone-derived α-amino acids, which were found to exist as planar and twisted rotamers, resulting in dual emission fluorescent properties.^11^ Based on this work, we proposed that a fluorescent α-amino acid bearing a poly aromatic side-chain with multiple rotation sites, which can adopt planar and twisted conformations could act as a viscosity probe. It was envisaged that the generation of a chromophore with an electron rich aryl ring conjugated to an electron deficient heterocycle as the amino acid side chain would possess significant charge transfer and exist in planar or twisted conformations. We previously described fluorescent α-amino acids with pyridine- and pyrimidine-derived side chains that displayed strong intramolecular charge transfer (ICT) fluorescent properties.^12^ Following this previous work, we proposed that triaryl amino acids **5**, that are based on a π-deficient pyridine side chain with multiple rotation sites could exist as twisted and planar rotamers and therefore possess sensitivity to viscosity (Figure 1c). Herein, we now report the synthesis and photophysical properties of novel pyridine-based α-amino acids that display strong, charge transfer based fluorescence. As well as exhibiting solvatochromism, we also demonstrate that the fluorescence of these α-amino acids can be controlled by viscosity through the interconversion between twisted and planar states.

It was proposed that triaryl α-amino acids **5** could be accessed in two stages from an enone-derived α-amino acid (Figure 1c). Initially, the first stage would generate the key pyridine ring using a Lewis acid-catalyzed hetero-Diels–Alder reaction and a modified Knoevenagel–Stobbe process. The second stage would then involve regioselective activation of the *p*-methoxyphenyl motif and subsequent arylation. A key objective was to incorporate arenes with different electronics to probe the photophysical properties of the triaryl chromophore. The first stage of the synthesis of amino acids **5** focused on the preparation of biaryl intermediate **10** (Scheme 1). This was prepared as previously described by us but with some key modifications.^12a^ Initially, enone-derived α-amino acid **9** was synthesized in four steps from l-aspartic acid derivative **6**.^13^ Regioselective reaction of aspartic acid diester **6** with the anion of dimethyl methylphosphonate gave β-ketophosphonate ester **7** in 92% yield. The trityl group was replaced with the smaller Cbz-protecting group by treatment of **7** with TFA and then reaction with benzyl chloroformate under standard conditions. Horner–Wadsworth–Emmons reaction of phosphonate ester **8** with *p*-methoxybenzaldehyde gave *E*-enone **9** as the sole product in 71% yield.^13^ Hetero-Diels–Alder reaction of enone **9** with ethyl vinyl ether using Yb(fod)_3_ as a Lewis acid catalyst^14^ gave the dihydropyran. Using the dihydropyran intermediate as a latent 1,5-dicarbonyl compound, reaction with hydroxylamine via a Knoevenagel–Stobbe reaction gave pyridine **10** in 63% yield over the two-steps.^15,16^ To complete the synthesis of the triaryl side chain, an *ortho*-halogenation, followed by a cross-coupling reaction was proposed. We have previously reported the use of the super Lewis acid, iron(III) triflimide for the regioselective halogenation of arenes.^17^ This reaction was investigated for the bromination of **10**. On optimization, the use of Fe(NTf_2_)_3_ (8 mol %) resulted in a fast reaction (4 h) and the preparation of **11** in 80% yield. Suzuki–Miyaura reaction of **11** with various boronic acids allowed the incorporation of electron-rich (**12a**), electron-deficient (**12b**) and highly conjugated arenes (**12c**) in 53–71% yields.^18^ Ester hydrolysis at room temperature using cesium carbonate, followed by acid-mediated removal of the Cbz-protecting group gave amino acids **5a**–**5c** in high yields. Overall, this straightforward approach permitted the efficient, late-stage incorporation of the final arene ring, allowing access to electronically diverse amino acids for assessment.

**Scheme 1:**
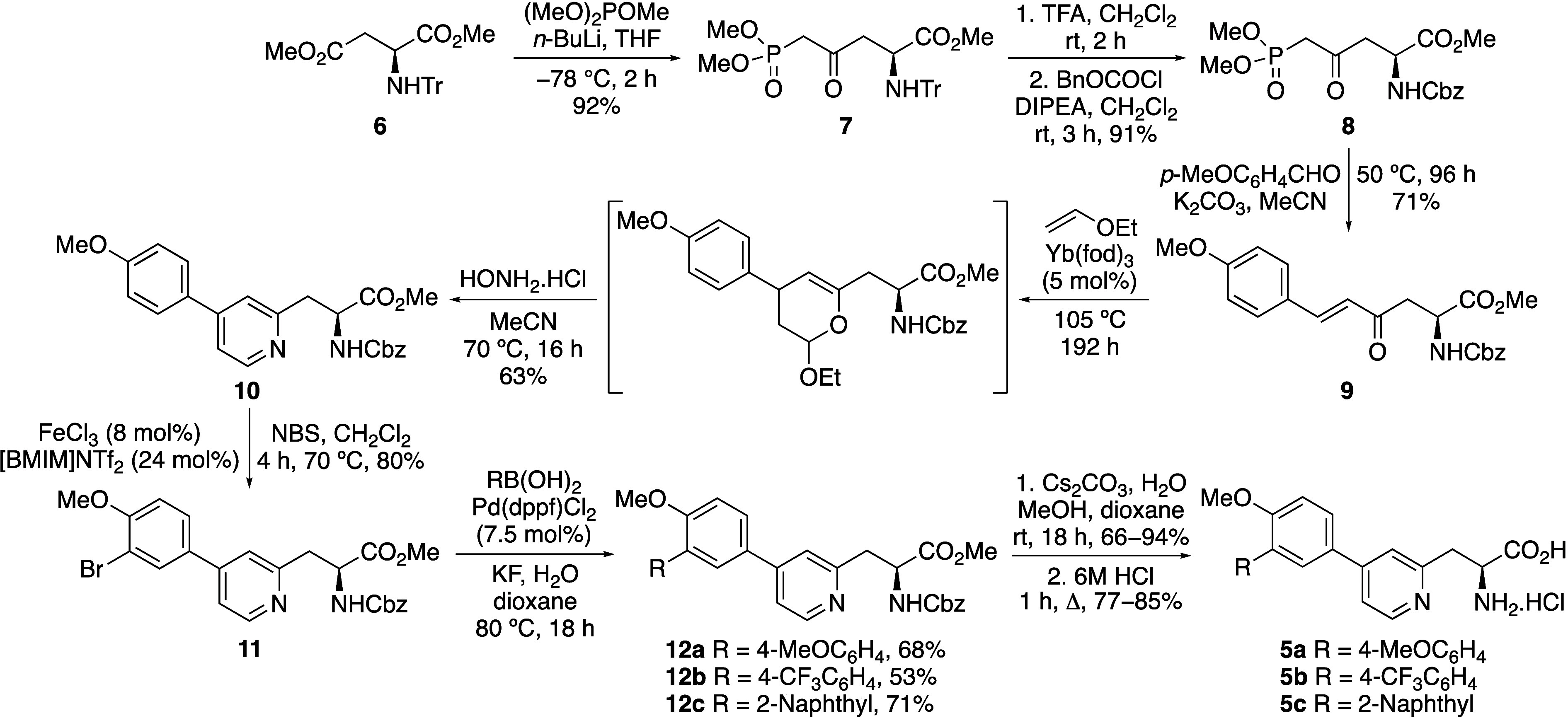
Synthesis of β-Pyridyl α-Amino Acids **5a**–**c**

The photoluminescent properties of novel β-pyridyl α-amino acids **5a**–**c** were then measured (Table 1).^19,20^ The UV/vis absorption and emission spectra were recorded at a concentration of 5 μM in MeOH. Although amino acids **5a**–**c** displayed absorption bands at similar wavelength (288–304 nm) and red-shifted in relation to natural α-amino acids (see SI), the emission properties were very different. Amino acid **5a** bearing an electron-rich 4-methoxyphenyl group showed strong fluorescence with an emission maximum at 445 nm (Figure 2a), a MegaStokes shift of 10,423 cm^–1^ and a quantum yield of 0.13. Amino acid **5b** with an electron-deficient 4-trifluoromethylphenyl moiety also displayed strong fluorescence with a quantum yield of 0.20 and was found to possess a smaller Stokes shift of 6711 cm^–1^, with a main emission band at 357 nm. Amino acid **5c** with a 2-naphthyl side chain exhibited weaker fluorescence with a quantum yield of 0.08 and multiple emission bands with maxima at 357 and 495 nm, likely due to the existence of both locally excited (LE) and twisted intramolecular charge transfer (TICT) states.

**Figure 2:**
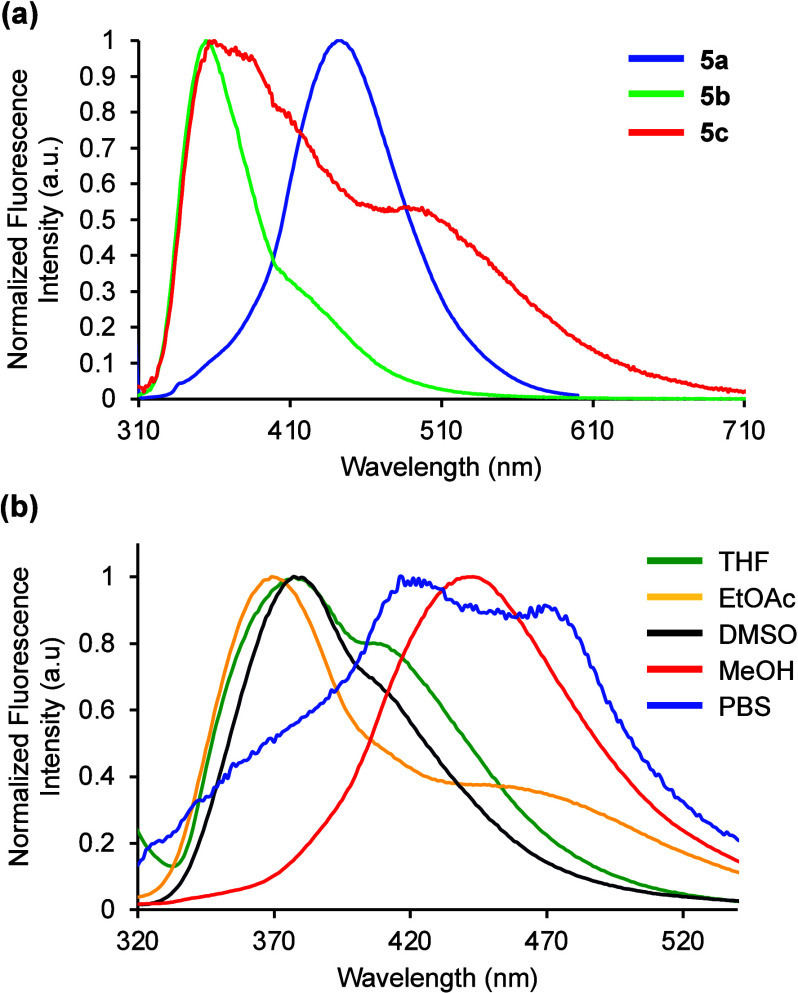
(a) Emission spectra of **5a**–**5c** (5 μM in MeOH). (b) Emission spectra of **5a** (5 μM) in various solvents.

**Table 1:** Photophysical Data of α-Amino Acids^a^

amino acid	λ_Abs_ (nm)	ε (cm^–1^ M^–1^)	λ_Em_ (nm)	Stokes shift (cm^–1^)	Φ_F_^b^
**5a**	304	19304	445	10423	0.13
**5b**	288	19316	357	6711	0.20
**5c**	295	20177	357, 495	5887, 13696	0.08

a Spectra were recorded at 5 μM in MeOH.

b Quantum yields (Φ_F_) were determined in MeOH using l-trp as a standard.

As amino acid **5a** showed the most promising properties of strong fluorescence, red-shifted emission and a large Stokes shift, this analogue was further evaluated. To demonstrate that the triaryl structure of **5a** possessed charge transfer properties that could potentially exist in a TICT state required for viscosity sensing, solvatochromism and DFT studies were conducted. From these studies, amino acid **5a** was found to be highly sensitive to solvent polarity, with a significant bathochromic shift of emission on increasing polarity (Figure 2b). In relatively nonpolar solvents such as EtOAc and THF, an emission maximum at 370 nm was observed, while in more polar solvents such as MeOH, and phosphate-buffered saline (PBS), emission was found at 445 and 470 nm, respectively. The solvatochromism of amino acid **5a** was further verified by a Lippert-Mataga plot (see SI), which showed a linear correlation between the Stokes shifts versus solvent orientation polarizability.^19,21^ These results confirm the design of the triaryl motif as a charge transfer chromophore, which on excitation is stabilized by more polar solvents. Further evidence for the charge transfer character of amino acid **5a** was provided by DFT calculations. Initially, an energy minimized model of **5a** was optimized in methanol with the ωB97X-D^22^ and def2-TZVP basis set,^23^ which confirmed a twisted conformation (Figure 3a). Frontier molecular orbital analysis at the same level of theory allowed visualization of the highest occupied molecular orbital (HOMO) and lowest unoccupied molecular orbital (LUMO) states. The electron density of the HOMO (Figure 3b) was found localized on the electron-rich 4-MeO-substituted rings, while the electron density contribution for the LUMO was focused on the pyridine ring (Figure 3c). This calculated electronic structure is consistent with the emission properties of amino acid **5a**. Electron donation from the 4-MeO-aryl rings through to the π-deficient pyridine ring results in a charge-separated excited state that on de-excitation produces emission at longer wavelengths. Combined, the solvatochromic and DFT studies verified the ICT character of amino acid **5a** and the potential of this fluorophore to sense changes in viscosity.

**Figure 3:**
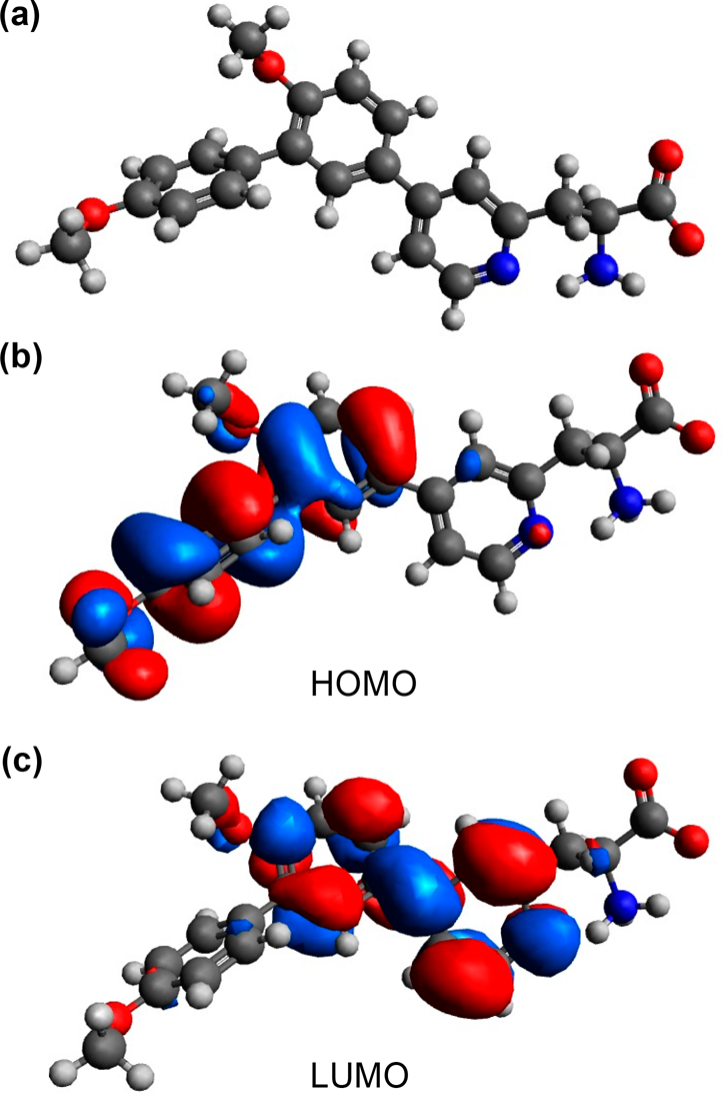
(a) Energy minimized DFT structure of **5a** using ωB97X-D and def2-TZVP basis sets. (b) Calculated HOMO isosurface plot from the DFT model. (c) Calculated LUMO isosurface plot from the DFT model.

Having explored the photophysical properties of amino acid **5a** and established the charge transfer character of the fluorophore, viscosity studies were next performed. Absorption and emission spectra were recorded in MeOH (viscosity, η = 0.59 mPa s) with increasing quantities of ethylene glycol (η = 13.5 mPa s).^24^ In contrast to the absorption spectra, which were found to be independent of viscosity (see SI), the emission spectra displayed significant changes (Figure 4a). In general, as the viscosity of the solution increased, the intensity of the major emission band at 445 nm decreased, with concurrent amplification of a minor band at 315 nm. We propose that these two distinct emission bands are derived from rotational conformers. At low viscosity in MeOH, the triaryl side chain of amino acid **5a** adopts a twisted conformation (Figure 4b), that on excitation relaxes to a TICT state, generating emission at longer wavelength (445 nm). As the viscosity increases to 100% ethylene glycol, the fluorophore is forced into a more planar conformation, in which emission at shorter wavelength (315 nm) from the LE state is observed. To verify these results were due to changes in viscosity rather than other solvent properties, further factors were considered. This effect is not polarity-dependent as ethylene glycol is more polar than MeOH and thus, a new emission band would be expected to occur at longer wavelength. The appearance of blue-shifted bands on changing solvents can be caused by aggregation,^25,26^ and therefore, a study of this nature was conducted. The emission spectra of amino acid **5a** was recorded at various concentrations in MeOH (Figure 4c).^27^ A 10-fold increase in concentration (50 μM), resulted in a small hypsochromic shift of the main band to 418 nm and the appearance of a second peak at longer wavelength (563 nm). Increasing the concentration by 40- and 100-fold (200 and 500 μM), generated similar emission spectra with decreasing intensity. The new red-shifted peak is likely due to formation of aggregates, while at high concentrations, aggregation-induced quenching is observed. In comparison with the viscosity study (Figure 4a), the aggregation spectra are very different and show no emission at 315 nm from the proposed LE state. These results suggest that the significant difference in fluorescence emission observed on changing from MeOH to glycol is viscosity-dependent, which is controlled by the twisted and planar conformational isomers of amino acid **5a**.

**Figure 4:**
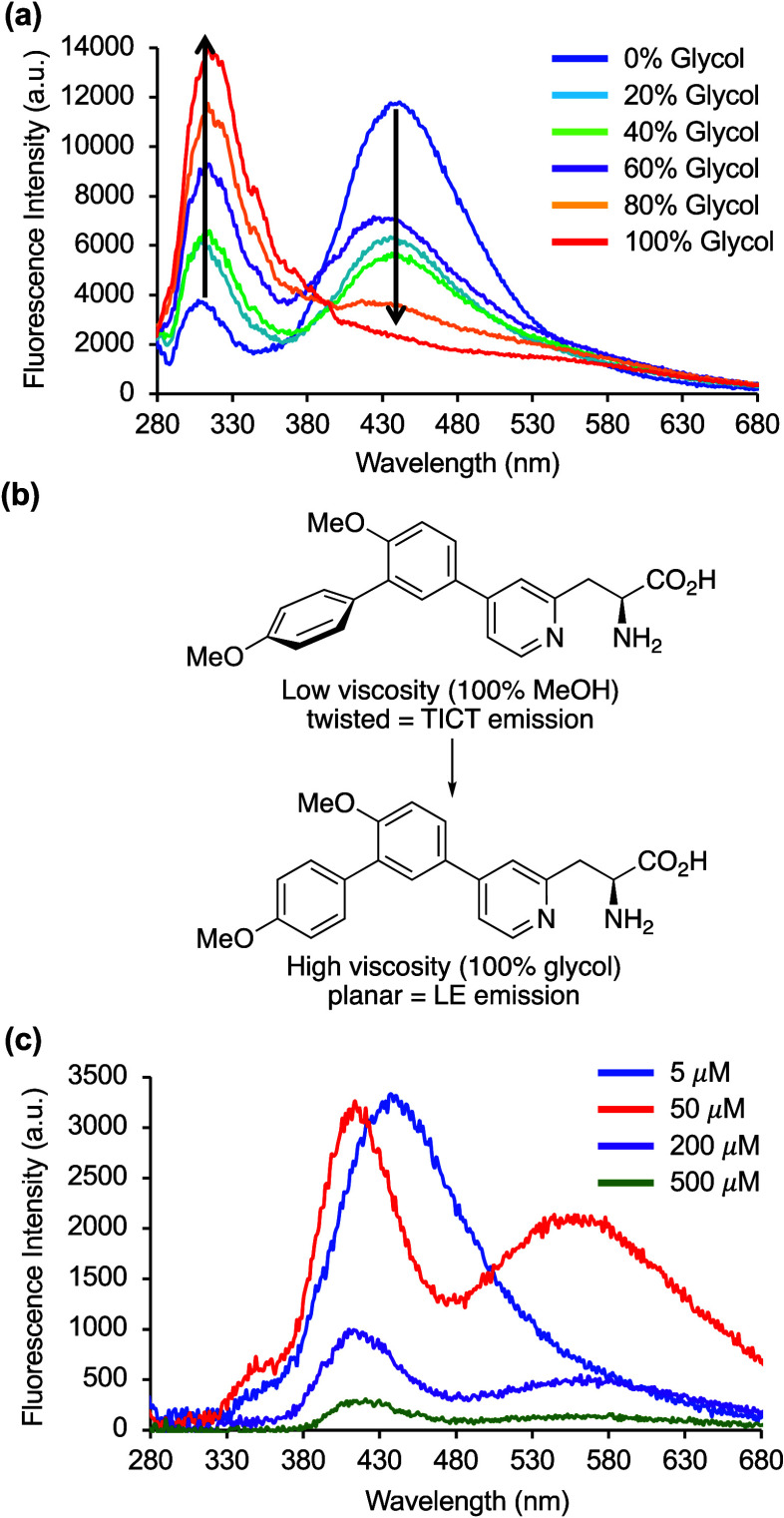
(a) Emission spectra of **5a** (5 μM) in ethylene glycol–MeOH mixtures. (b) Twisted and planar rotamers that result in TICT and LE emission, respectively. (c) Emission spectra of **5a** at various concentrations in MeOH.

In summary, we report the development of a novel α-amino acid that exhibits rotamer-controlled fluorescence. The amino acids were synthesized in two stages, involving preparation of the β-pyridyl motif, followed by late-stage introduction of the final aryl ring. Photophysical studies allowed the discovery of amino acid **5a** that displayed strong TICT emission, which was confirmed by a solvatochromism study and DFT calculations. Viscosity studies demonstrated that the emission of **5a** could be controlled by the conformation of the triaryl fluorophore. At low viscosity and in a twisted conformation, emission occurred from the TICT state at long wavelength, while at high viscosity and in a more planar conformation, emission from the LE state was found at shorter wavelength. Work is underway to develop the potential of these amino acids as viscosity probes for chemical biology applications.

## Data Availability

The data underlying this study are available in the published article and its Supporting Information.

## Supporting Information Available

Experimental procedures, characterization data, photophysical data, and NMR spectra of all compounds (PDF)
