# Supplementary material for: Synthesis of β-Pyridyl α-Amino Acids: Conformationally Sensitive Charge Transfer-Based Fluorophores
Source: Org Lett. 2024 Jun 12;26(25):5391–5. doi: 10.1021/acs.orglett.4c01951 (PMC11217948; doi:10.1021/acs.orglett.4c01951)

Supporting Information for:

Synthesis of β-Pyridyl α-Amino Acids: Conformationally Sensitive Charge Transfer-Based Fluorophores

*Leanne M. Riley, Olivia Marshall, Alexander H. Harkiss, Hans M. Senn and Andrew Sutherland**

School of Chemistry, The Joseph Black Building, University Avenue, University of Glasgow, Glasgow, G12 8QQ, United Kingdom.

Table of Contents

1. General Experimental	S2–S3
2. Experimental Procedures and Spectroscopic Data for all Compounds	S3–S11
3. Computational Details	S12–S13
4. References	S14
5. Photophysical Data for α-Amino Acids	S15–S20
6. ^{1}H and ^{13}C NMR Spectra for all Compounds	S21–S42

1. General Experimental

All reagents and starting materials were obtained from commercial sources and used as received. Dimethyl (2*S*)-2-(tritylamino)butandioate (**6**) was prepared as previously described.[1] Dry solvents were purified using a PureSolv 500 MD solvent purification system. All reactions were performed open to air unless otherwise mentioned. All reactions performed at elevated temperatures were heated using an oil bath. Brine refers to a saturated aqueous solution of sodium chloride. Flash column chromatography was carried out using Merck Millipore matrix silicagel 60 (40–63 μM). Merck aluminium-backed plates pre-coated with silica gel 60 (UV_{254}) were used for thin-layer chromatography and visualized with a UV lamp. ^{1}H NMR and ^{13}C NMR spectra were recorded on a Bruker DPX 400 or 500 spectrometer, with chemical shift values in ppm relative to tetramethylsilane (δ_H 0.00 and δ_C 0.00), or for ^{1}H NMR, relative to residual chloroform (δ_H 7.26) or methanol (δ_H 3.31) as standard. For ^{13}C NMR the chemical shifts are reported relative to the central resonance of $CDCl_3$ (δ_C 77.2) or CD_3OD (δ_C 49.0) as standard. Carbon assignments are based on two-dimensional HMBC and DEPT experiments. Mass spectra were obtained either using a JEOL JMS-700 spectrometer for EI and CI, and Bruker Microtof-q or Agilent 6125B for ESI. Infrared spectra were obtained neat using a Shimadzu IR Prestige-21 spectrometer or Shimadzu 8400S spectrometer; wavenumbers are indicated in cm^{-1}. Melting points were determined on either a Reichert platform melting point apparatus or Stuart Scientific melting point apparatus. Optical rotations were determined as solutions irradiating with the sodium D line (λ = 589 nm) using an Autopol V polarimeter. $[\alpha]_D$ values are given in units 10^{-1} deg cm^2 g^{-1}. Absorption and emission data were recorded on one of two instruments:

1. UV-Vis spectra were recorded on a Pekin Elmer Lamda 25 instrument. Fluorescence spectra were recorded on a Shimadzu RF-5301PC spectrofluorophotometer. Emission data were measured using excitation and emission bandpass filters of 3 nm.

2. Both UV-Vis spectra and fluorescence spectra were recorded on a Horiba Duetta Fluorescence and Absorbance spectrometer. Absorbance spectra were recorded with an integration time of 0.05 s, and a band pass of 5 nm. Fluorescence spectra were recorded with excitation and emission band pass of 5 nm, an integration time of 0.1 s, and with detector accumulations set to 1. Respective standard samples were recorded with the same parameters.

Quantum yields were determined using L-tryptophan (Φ = 0.14 in water) as the standard reference.[2] The integrated fluorescence intensity of each compound was determined from the emission spectra given. Measurements were performed at five different concentrations. Concentrations were chosen to ensure the absorption value was below 0.1 to avoid re-absorption effects. Integrated fluorescence

intensity was plotted as a function of the measured absorbance and a linear fit was calculated. The resultant gradient was then used to calculate the quantum yield, using the equation below:

$$\phi_x = \phi_{ST}\left(\frac{Grad_{ST}}{Grad_x}\right)\left(\frac{\eta_x^2}{\eta_{ST}^2}\right)$$

Subscript *ST* signifies the quantities associated with the quantum yield standard. Subscript X signifies the quantities associated with the novel compound. $Grad_X$ is the determined gradient associated with the novel compound. $Grad_{ST}$ is the determined gradient associated with quantum yield standard. η is the refractive index of the solvent used in the fluorescence measurements. η = 1.333 for water, 1.361 for ethanol and 1.331 for methanol.

2. Experimental Procedures and Spectroscopic Data for all Compounds

Methyl (2*S*)-5-(dimethoxyphosphoryl)-4-oxo-2-(tritylamino)pentanoate (7)[3]

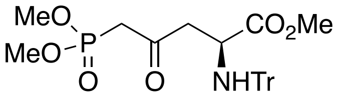

A solution of dimethyl methylphosphonate (2.95 mL, 27.3 mmol) in tetrahydrofuran (50 mL) was cooled to –78 °C under an argon atmosphere. *n*-Butyl lithium (2.5 M in hexane, 11.4 mL, 28.5 mmol) was added dropwise and the reaction mixture stirred for 1 h. In a separate reaction vessel, a solution of dimethyl (2*S*)-2-(tritylamino)butandioate (**6**) (5.00 g, 12.4 mmol) in tetrahydrofuran (100 mL) was cooled to –78 °C and then the dimethyl methylphosphonate/*n*-butyl lithium solution was cannulated into the flask and the reaction mixture stirred at –78 °C for 2 h to give a yellow solution. The reaction was quenched with a saturated solution of ammonium chloride (3 mL) and allowed to warm to room temperature. The mixture was concentrated *in vacuo*. The resulting residue was diluted with ethyl acetate (100 mL), washed with water (2 × 100 mL), brine (100 mL) then dried ($MgSO_4$) and concentrated *in vacuo*. Purification by flash column chromatography, eluting with 75% ethyl acetate in petroleum ether gave methyl (2*S*)-5-(dimethoxyphosphoryl)-4-oxo-2-(tritylamino)pentanoate (**7**) as a colorless solid (5.61 g, 92%). Mp 117–118 °C (lit.[3] 117–118.5 °C); $[\alpha]_D^{24}$ +31.1 (*c* 1.0, $CHCl_3$); ^{1}H NMR (400 MHz, $CDCl_3$) δ 7.47 (d, J = 7.7 Hz, 6H), 7.26 (t, J = 7.7 Hz, 6H), 7.21–7.15 (m, 3H), 3.79 (s, 3H), 3.76 (s, 3H), 3.73–3.65 (m, 1H), 3.29 (s, 3H), 3.06 (d, $^2J_{H-P}$ = 22.7 Hz, 2H), 2.95–2.85 (m, 2H), 2.78 (dd, J = 16.7, 6.9 Hz, 1H); ^{13}C{^{1}H} NMR (101 MHz, $CDCl_3$) δ 199.3, 174.0, 145.7, 128.8, 127.9, 126.6, 71.3, 53.1, 53.0, 52.9, 52.0, 48.8, 41.8 (d, $^1J_{C-P}$ = 128 Hz); MS (CI) *m/z* 496 (M + H$^+$, 1), 301 (5), 254 (90), 243 (100), 237 (55), 167 (45).

Methyl (2*S*)-2-[(benzyloxycarbonyl)amino]-5-(dimethoxyphosphoryl)-4-oxopentanoate (8)

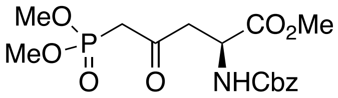

To a solution of methyl (2*S*)-5-(dimethoxyphosphoryl)-4-oxo-2-(tritylamino)pentanoate (**7**) (4.70 g, 9.50 mmol) in dichloromethane (150 mL) was added trifluoroacetic acid (1.50 mL, 19.0 mmol). The reaction mixture was stirred at room temperature for 1 h before concentration *in vacuo*. The resulting residue was dissolved in chloroform (5 mL) and petroleum ether (40–60) was added until an orange oil formed, at which point the solvent was decanted off. The resulting oil was dissolved in dichloromethane (150 mL) and *N*,*N*-diisopropylethylamine (4.20 mL, 23.8 mmol) was added, followed by benzyl chloroformate (2.00 mL, 14.3 mmol). The reaction mixture was stirred at room temperature for 3 h, before diluting with water (50 mL). The mixture was then extracted with dichloromethane (3 × 50 mL), dried ($MgSO_4$) and concentrated *in vacuo*. Purification by flash column chromatography, eluting with 3% methanol in dichloromethane gave methyl (2*S*)-2-[(benzyloxycarbonyl)amino]-5-(dimethoxyphosphoryl)-4-oxopentanoate (**8**) as an orange oil (3.28 g, 91%). IR (neat) 3265, 2924, 1716, 1526, 1252, 1213, 1024, 810, 742, 700 cm^{-1}; $[\alpha]_D^{24}$ +22.5 (*c* 1.0, $CHCl_3$); ^1H NMR (400 MHz, $CDCl_3$) δ 7.37–7.31 (m, 5H), 5.74 (d, J = 8.5 Hz, 1H), 5.11 (s, 2H), 4.59 (ddd, J = 8.5, 4.7, 4.3 Hz, 1H), 3.77 (d, $^3J_{H\text{–}P}$ = 0.6 Hz, 3H), 3.74–3.73 (m, 6H), 3.35 (dd, J = 18.5, 4.7 Hz, 1H), 3.17 (dd, J = 18.5, 4.3 Hz, 1H), 3.09 (d, $^2J_{H\text{–}P}$ = 22.7 Hz, 2H); $^{13}C\{^1H\}$ NMR (101 MHz, $CDCl_3$) δ 199.7 (d, $^2J_{C\text{–}P}$ = 6.0 Hz), 171.2, 156.0, 136.2, 128.5, 128.2, 128.1, 67.0, 53.2 (d, $^2J_{C\text{–}P}$ = 7.2 Hz), 53.1 (d, $^2J_{C\text{–}P}$ = 7.2 Hz), 52.7, 49.8, 45.6, 41.5 (d, $^1J_{C\text{–}P}$ = 128 Hz); MS (ESI) *m/z* 410 (M + Na^+, 100); HRMS (ESI) *m/z*: $[M + Na]^+$ Calcd for $C_{16}H_{22}NO_8PNa$ 410.0975; Found 410.0966.

Methyl (2*S*,5*E*)-2-[(benzyloxycarbonyl)amino]-6-(4'-methoxyphenyl)-4-oxohex-5-enoate (9)[4]

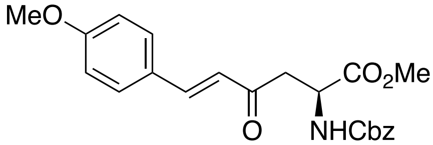

Methyl (2*S*)-2-[(benzyloxycarbonyl)amino]-5-(dimethoxyphosphoryl)-4-oxopentanoate (**8**) (0.500 g, 1.29 mmol) was dissolved in anhydrous acetonitrile (10 mL) and potassium carbonate (0.196 g, 1.42 mmol) was added. The mixture was stirred at room temperature for 0.5 h followed by addition of 4-methoxybenzaldehyde (0.340 mL, 2.58 mmol). The temperature was increased to 50 °C and the mixture was stirred for 72 h. Once the reaction was complete, the solution was concentrated *in vacuo* and the resulting residue was dissolved in ethyl acetate (20 mL) and washed with water (2 × 15 mL), brine (15 mL), dried ($MgSO_4$) and concentrated *in vacuo*. Purification by flash column

chromatography, eluting with 50% diethyl ether in petroleum ether (40–60) gave methyl (2*S*,5*E*)-2-[(benzyloxycarbonyl)amino]-6-(4'-methoxyphenyl)-4-oxohex-5-enoate (**9**) as a yellow oil (0.368 g, 71%). Spectroscopic data were consistent with the literature.[4] $[\alpha]_D^{29}$ +30.3 (*c* 1.0, $CHCl_3$); ^1H NMR (400 MHz, $CDCl_3$) δ 7.52 (d, *J* = 16.2 Hz, 1H), 7.49 (d, *J* = 8.8 Hz, 2H), 7.38−7.27 (m, 5H), 6.92 (d, *J* = 8.8 Hz, 2H), 6.58 (d, *J* = 16.2 Hz, 1H), 5.88 (d, *J* = 8.5 Hz, 1H), 5.12 (s, 2H), 4.67 (dt, *J* = 8.5, 4.2 Hz, 1H), 3.85 (s, 3H), 3.75 (s, 3H), 3.47 (dd, *J* = 17.9, 4.2 Hz, 1H), 3.23 (dd, *J* = 17.9, 4.2 Hz, 1H); $^{13}C\{^1H\}$ NMR (101 MHz, $CDCl_3$) δ 197.4, 171.8, 162.1, 156.2, 144.0, 136.4, 130.4, 128.6, 128.2, 128.1, 126.9, 123.4, 114.6, 67.1, 55.6, 52.8, 50.3, 42.3; MS (EI) *m/z* 397 (M^+, 90), 336 (10), 289 (19), 262 (19), 243 (45), 182 (34), 161 (100), 91 (32).

Methyl (2*S*)-2-[(benzyloxycarbonyl)amino]-3-[4'-(4"-methoxyphenyl)pyridin-2'-yl]propanoate (10)[5]

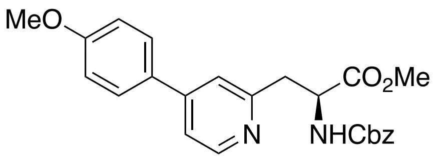

To a solution of methyl (2*S*,5*E*)-2-[(benzyloxycarbonyl)amino]-6-(4'-methoxyphenyl)-4-oxohex-5-enoate (**9**) (1.00 g, 2.52 mmol) in ethyl vinyl ether (12 mL) was added tris(6,6,7,7,8,8,8-heptafluoro-2,2-dimethyl-3,5-octanedionato)ytterbium (0.134 g, 0.126 mmol) in a sealed tube. The tube was then purged with argon, sealed and the reaction mixture was stirred at 110 °C for 168 h. The mixture was then allowed to cool to room temperature and concentrated *in vacuo*. The reaction mixture was washed through a silica plug eluting with 20% ethyl acetate in petroleum ether (40–60) and gave the dihydropyran. This was used for the next step without further purification. The dihydropyran was then reacted with hydroxylamine hydrochloride (0.740 g, 10.7 mmol) in acetonitrile (80 mL) at 70 °C for 16 h. Purification by flash column chromatography, eluting with 1% methanol in dichloromethane gave methyl (2*S*)-2-[(benzyloxycarbonyl)amino]-3-[4'-(4"-methoxyphenyl)pyridin-2'-yl]propanoate (**10**) (0.576 g, 63%) as a yellow oil. Spectroscopic data were consistent with the literature.[5] $[\alpha]_D^{25}$ +23.0 (*c* 0.8, $CHCl_3$); ^1H NMR (500 MHz, $CDCl_3$) δ 8.47 (d, *J* = 5.2 Hz, 1H), 7.55 (d, *J* = 8.7 Hz, 2H), 7.35–7.29 (m, 7H), 6.99 (d, *J* = 8.7 Hz, 2H), 6.36 (d, *J* = 8.2 Hz, 1H), 5.13 (d, *J* = 12.3 Hz, 1H), 5.09 (d, *J* = 12.3 Hz, 1H), 4.80 (dt, *J* = 8.2, 5.2 Hz, 1H), 3.86 (s, 3H), 3.70 (s, 3H), 3.42 (dd, *J* = 14.9, 5.2 Hz, 1H), 3.33 (dd, *J* = 14.9, 5.2 Hz, 1H); $^{13}C\{^1H\}$ NMR (126 MHz, $CDCl_3$) δ 172.3, 160.8, 157.5, 156.3, 149.6, 148.8, 136.6, 130.4, 128.6, 128.3, 128.2, 121.2, 119.6, 114.7, 67.0, 55.6, 53.6, 52.5, 39.2; MS (ESI) *m/z* 443 (M + Na^+, 100).

Methyl (2*S*)-2-[(benzyloxycarbonyl)amino]-3-[4'-(3"-bromo-4"-methoxyphenyl)pyridin-2'-yl]propanoate (11)

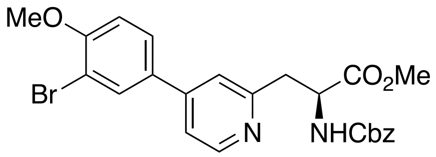

Iron(III) chloride (0.0250 g, 0.156 mmol) in 1-butyl-3-methylimidazolium bis-(trifluoromethanesulfonyl)imide (0.140 mL, 0.468 mmol) was stirred for 0.5 h at room temperature. This solution was added to *N*-bromosuccinimide (1.04 g, 5.85 mmol) and methyl (2*S*)-2-[(benzyloxycarbonyl)amino]-3-[4'-(4"-methoxyphenyl)pyridin-2'-yl]propanoate (**10**) (0.820 g, 1.95 mmol) in dichloromethane (3 mL). The reaction mixture was heated to 70 °C for 4 h. Upon completion, the reaction mixture was filtered through Celite® and concentrated *in vacuo*. Purification by flash column chromatography, eluting with 10% acetonitrile in dichloromethane gave methyl (2*S*)-2-[(benzyloxycarbonyl)amino]-3-[4'-(3"-bromo-4"-methoxyphenyl)pyridin-2'-yl]propanoate (**11**) (0.774 g, 80%) as a yellow oil. IR (neat) 3345, 2953, 1717, 1600, 1504, 1454, 1287, 1258, 1213, 1053, 812 cm^{-1}; $[\alpha]_D^{19}$ +11.1 (*c* 1.0, $CHCl_3$); ^1H NMR (500 MHz, $CDCl_3$) δ 8.48 (d, J = 5.2 Hz, 1H), 7.80 (d, J = 2.2 Hz, 1H), 7.51 (dd, J = 8.6, 2.2 Hz, 1H), 7.35–7.26 (m, 7H), 6.97 (d, J = 8.6 Hz, 1H), 6.35 (d, J = 8.3 Hz, 1H), 5.12 (d, J = 12.5 Hz, 1H), 5.09 (d, J = 12.5 Hz, 1H), 4.80 (dt, J = 8.3, 5.2 Hz, 1H), 3.94 (s, 3H), 3.70 (s, 3H), 3.42 (dd, J = 14.9, 5.2 Hz, 1H), 3.33 (dd, J = 14.9, 5.2 Hz, 1H); $^{13}C\{^1H\}$ NMR (126 MHz, $CDCl_3$) δ 172.2, 157.7, 156.8, 156.2, 149.7, 147.3, 136.4, 131.9, 131.6, 128.6, 128.2, 127.2, 121.0, 119.4, 112.5, 112.3, 67.0, 56.5, 53.4, 52.5, 39.1; MS (ESI) *m/z* 521 (M + Na^+, 100): HRMS (ESI) *m/z*: $[M + Na]^+$ Calcd for $C_{24}H_{23}{}^{79}BrN_2O_5Na$ 521.0683; Found 521.0682.

Methyl (2*S*)-2-[(benzyloxycarbonyl)amino]-3-[4'-(4",4'"-dimethoxy-[1",1'"-biphenyl]-3"-yl)pyridin-2'-yl]propanoate (12a)

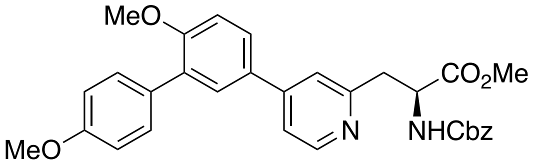

To a microwave vial containing 4-methoxyphenylboronic acid (0.0780 g, 0.510 mmol), potassium fluoride (0.0600 g, 1.02 mmol) and [1,1'-bis(diphenylphosphino)ferrocene]palladium(II) dichloride-dichloromethane complex (0.0255 g, 0.0210 mmol) was added to a solution of methyl (2*S*)-2-[(benzyloxycarbonyl)amino]-3-[4'-(3"-bromo-4"-methoxyphenyl)pyridin-2'-yl]propanoate (**11**) (0.170 g, 0.340 mmol) in 1,4-dioxane (3.0 mL) and water (0.4 mL). The reaction mixture was

degassed, and then stirred at 80 °C for 20 h. The reaction mixture was filtered through Celite® and washed with ethyl acetate (50 mL). The filtrate was washed with water (50 mL) and brine (50 mL), dried ($MgSO_4$) and concentrated *in vacuo* to give the crude product. Purification by flash column chromatography, eluting with 20% acetonitrile in dichloromethane gave methyl (2*S*)-2-[(benzyloxycarbonyl)amino]-3-[4'-(4",4'"-dimethoxy-[1",1'"-biphenyl]-3"-yl)pyridin-2'-yl]propanoate (**12a**) as a yellow oil (0.122 g, 68%). IR (neat) 3304, 2951, 1715, 1693, 1601, 1499, 1455, 1243, 1023, 831 cm^{-1}; $[\alpha]_D^{22}$ +16.7 (*c* 1.0, $CHCl_3$); ^1H NMR (500 MHz, $CDCl_3$) δ 8.47 (d, *J* = 5.1 Hz, 1H), 7.56–7.52 (m, 2H), 7.49 (d, *J* = 8.8 Hz, 2H), 7.36–7.25 (m, 7H), 7.04 (d, *J* = 9.2 Hz, 1H), 6.98 (d, *J* = 8.8 Hz, 2H), 6.38 (d, *J* = 8.3 Hz, 1H), 5.10 (s, 2H), 4.80 (dt, *J* = 8.3, 5.1 Hz, 1H), 3.86 (s, 3H), 3.85 (s, 3H), 3.69 (s, 3H), 3.42 (dd, *J* = 14.9, 5.1 Hz, 1H), 3.33 (dd, *J* = 14.9, 5.1 Hz, 1H); $^{13}C\{^1H\}$ NMR (126 MHz, $CDCl_3$) δ 172.2, 159.0, 157.5, 157.4, 156.1, 149.6, 148.6, 136.4, 131.1, 130.6, 130.3, 130.2, 129.3, 128.5, 128.1, 126.8, 121.1, 119.5, 113.7, 111.7, 66.9, 55.8, 55.3, 53.4, 52.4, 39.0; MS (ESI) *m/z* 527 (M + H^+, 100); HRMS (ESI) *m/z*: $[M + H]^+$ Calcd for $C_{31}H_{30}N_2O_6H$ 527.2177; Found 527.2168.

Methyl (2*S*)-2-[(benzyloxycarbonyl)amino]-3-[4'-(3"-(4'"-trifluoromethylphenyl)-4"-methoxyphenyl)pyridin-2'-yl]propanoate (12b)

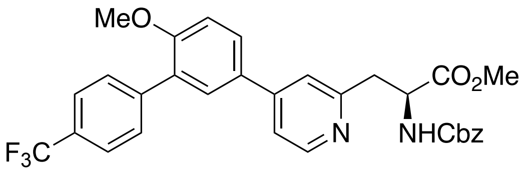

Methyl (2*S*)-2-[(benzyloxycarbonyl)amino]-3-[4'-(3"-(4'"-trifluoromethylphenyl)-4"-methoxyphenyl)pyridin-2'-yl]propanoate (**12b**) was synthesized as described above using 4-(trifluoromethyl)phenylboronic acid (0.0850 g, 0.450 mmol), potassium fluoride (0.0523 g, 0.900 mmol), [1,1'-bis(diphenylphosphino)ferrocene]palladium(II) dichloride-dichloromethane complex (0.0184 g, 0.0225 mmol) and methyl (2*S*)-2-[(benzyloxycarbonyl)amino]-3-[4'-(3"-bromo-4"-methoxyphenyl)pyridin-2'-yl]propanoate (**11**) (0.150 g, 0.300 mmol) in 1,4-dioxane (2.0 mL) and water (0.24 mL). Purification by flash column chromatography, eluting with 10% acetonitrile in dichloromethane gave methyl (2*S*)-2-[(benzyloxycarbonyl)amino]-3-[4'-(3"-(4'"-trifluoromethylphenyl)-4"-methoxyphenyl)pyridin-2'-yl]propanoate (**12b**) as an off-white solid (0.0900 g, 53%). Mp 127–129 °C; IR (neat) 3333, 2951, 1721, 1605, 1505, 1327, 1265, 1165, 1123, 1069, 756 cm^{-1}; $[\alpha]_D^{19}$ +16.4 (*c* 1.0, $CHCl_3$); ^1H NMR (400 MHz, $CDCl_3$) δ 8.49 (d, *J* = 4.9 Hz, 1H), 7.75–7.60 (m, 5H), 7.56 (d, *J* = 2.3 Hz, 1H), 7.39–7.25 (m, 7H), 7.09 (d, *J* = 8.6 Hz, 1H), 6.34 (d, *J* = 8.4 Hz, 1H), 5.11 (s, 2H), 4.81 (dt, *J* = 8.4, 5.1 Hz, 1H), 3.89 (s, 3H), 3.70 (s, 3H), 3.43 (dd, *J* =

14.9, 5.1 Hz, 1H), 3.34 (dd, J = 14.9, 5.1 Hz, 1H); $^{13}C\{^1H\}$ NMR (101 MHz, $CDCl_3$) δ 172.1, 157.6, 157.4, 156.1, 149.6, 148.2, 141.6, 136.4, 130.6, 130.0, 129.9, 129.4 (q, $^2J_{C-F}$ = 36.3 Hz), 129.4, 128.5, 128.08, 128.07, 128.0, 126.1 (q, $^3J_{C-F}$ = 3.7 Hz), 124.3 (q, $^1J_{C-F}$ = 272.0 Hz), 121.0, 119.5, 111.8, 66.9, 55.8, 53.4, 52.4, 39.1; MS (ESI) *m/z* 565 (M + H^+, 100); HRMS (ESI) *m/z*: $[M + H]^+$ Calcd for $C_{31}H_{27}F_3N_2O_5H$ 565.1945; Found 565.1942.

Methyl (2*S*)-2-[(benzyloxycarbonyl)amino]-3-[4'-(3"-(naphthalen-2'"-yl)-4"-methoxyphenyl)pyridin-2'-yl]propanoate (12c)

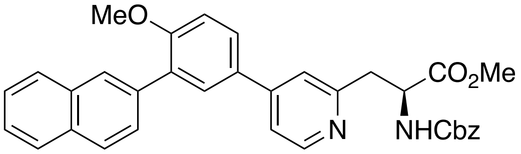

Methyl (2*S*)-2-[(benzyloxycarbonyl)amino]-3-[4'-(3"-(naphthalen-2'"-yl)-4"-methoxyphenyl)pyridin-2'-yl]propanoate (**12c**) was synthesized as described above using 2-naphthylboronic acid (0.0770 g, 0.451 mmol), potassium fluoride (0.0523 g, 0.900 mmol), [1,1'-bis(diphenylphosphino)ferrocene]palladium(II) dichloride-dichloromethane complex (0.0184 g, 0.0225 mmol) and methyl (2*S*)-2-[(benzyloxycarbonyl)amino]-3-[4'-(3"-bromo-4"-methoxyphenyl)pyridin-2'-yl]propanoate (**11**) (0.150 g, 0.300 mmol) in 1,4-dioxane (2.0 mL) and water (0.24 mL). Purification by flash column chromatography, eluting with 10% acetonitrile in dichloromethane gave methyl (2*S*)-2-[(benzyloxycarbonyl)amino]-3-[4'-(3"-(naphthalen-2'"-yl)-4"-methoxyphenyl)pyridin-2'-yl]propanoate (**12c**) as a yellow solid (0.116 g, 71%). Mp 100−103 °C; IR (neat) 3345, 2951, 1717, 1601, 1505, 1250, 1251, 752 cm^{-1}; $[\alpha]_D^{20}$ +12.4 (*c* 1.0, $CHCl_3$); ^1H NMR (400 MHz, $CDCl_3$) δ 8.50 (d, J = 5.1 Hz, 1H), 7.99 (br s, 1H), 7.93–7.85 (m, 3H), 7.73–7.67 (m, 2H), 7.62 (dd, J = 8.5, 2.1 Hz, 1H), 7.53–7.48 (m, 2H), 7.42–7.27 (m, 7H), 7.11 (d, J = 8.5 Hz, 1H), 6.37 (d, J = 8.1 Hz, 1H), 5.11 (s, 2H), 4.81 (dt, J = 8.1, 5.1 Hz, 1H), 3.90 (s, 3H), 3.71 (s, 3H), 3.44 (dd, J = 14.9, 5.1 Hz, 1H), 3.35 (dd, J = 14.9, 5.1 Hz, 1H); $^{13}C\{^1H\}$ NMR (101 MHz, $CDCl_3$) δ 172.2, 157.7, 157.5, 156.1, 149.6, 148.5, 136.4, 135.6, 133.4, 132.6, 131.5, 130.4, 129.8, 128.5, 128.22, 128.16, 128.1, 127.8, 127.7, 127.4, 127.3, 126.14, 126.08, 121.1, 119.5, 111.8, 66.9, 55.9, 53.4, 52.4, 39.0; MS (ESI) *m/z* 547 (M + H^+, 100); HRMS (ESI) *m/z*: $[M + H]^+$ Calcd for $C_{34}H_{30}N_2O_5H$ 547.2227; Found 547.2224.

(2*S*)-2-Amino-3-[4’-(4”,4”’-dimethoxy-[1”,1”’-biphenyl]-3”-yl)pyridin-2’-yl]propanoic acid hydrochloride (5a)

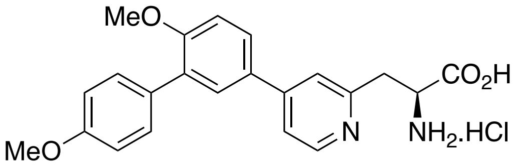

To a solution of methyl (2*S*)-2-[(benzyloxycarbonyl)amino]-3-[4’-(4”,4”’-dimethoxy-[1”,1”’-biphenyl]-3”-yl)pyridin-2’-yl]propanoate (**12a**) (0.100 g, 0.200 mmol) in a mixture of methanol (4 mL) and 1,4-dioxane (4 mL) was added a solution of caesium carbonate (0.0850 g, 0.260 mmol) in water (2 mL). The reaction mixture was stirred at room temperature for 20 h and then concentrated *in vacuo.* The resulting residue was dissolved in water (10 mL) and acidified to pH 1 with 2 M aqueous hydrochloric acid. The aqueous layer was extracted with dichloromethane (3 × 30 mL) and the combined organic layers were dried ($MgSO_4$) and concentrated *in vacuo* to give (2*S*)-2-[(benzyloxycarbonyl)amino]-3-[4’-(4”,4”’-dimethoxy-[1”,1”’-biphenyl]-3”-yl)pyridin-2’-yl]propanoic acid as a yellow solid (0.0830g, 66%) which was used for the next reaction without any further purification. (2*S*)-2-[(Benzyloxycarbonyl)amino]-3-[4’-(4”,4”’-dimethoxy-[1”,1”’-biphenyl]-3”-yl)pyridin-2’-yl]propanoic acid (0.0500 g, 0.103 mmol) was suspended in 6 M aqueous hydrochloric acid (5 mL) and heated under reflux for 1 h. The reaction mixture was cooled to room temperature and concentrated *in vacuo*. Purification by recrystallization from a mixture of methanol and diethyl ether gave (2*S*)-2-amino-3-[4’-(4”,4”’-dimethoxy-[1”,1”’-biphenyl]-3”-yl)pyridin-2’-yl]propanoic acid hydrochloride (**5a**) as a yellow solid (0.0350 g, 85%). Mp 175−180 °C (decomposition); IR (neat) 3389, 2835, 1743, 1632, 1596, 1479, 1268, 1245, 1180, 1021, 833 cm^{-1}; $[\alpha]_D^{22}$ +18.9 (*c* 0.2, MeOH); ^{1}H NMR (400 MHz, CD_3OD) δ 8.71 (d, J = 6.2 Hz, 1H), 8.46 (d, J = 1.0 Hz, 1H), 8.29 (dd, J = 6.2, 1.0 Hz, 1H), 8.05 (dd, J = 8.7, 2.3 Hz, 1H), 7.95 (d, J = 2.3 Hz, 1H), 7.51 (d, J = 8.8 Hz, 2H), 7.31 (d, J = 8.7 Hz, 1H), 6.99 (d, J = 8.8 Hz, 2H), 4.74 (dd, J = 8.2, 6.6 Hz, 1H), 3.93 (s, 3H), 3.84 (s, 3H), 3.75 (dd, J = 15.6, 6.6 Hz, 1H), 3.69 (dd, J = 15.6, 8.2 Hz, 1H); ^{13}C{^{1}H} NMR (101 MHz, CD_3OD) δ 169.9, 161.7, 160.7, 158.8, 151.7, 142.7, 133.3, 131.8, 131.3, 130.9, 130.0, 127.6, 125.3, 123.1, 114.6, 113.6, 56.5, 55.8, 52.9, 35.1; MS (ESI) *m/z* 379 (M + H^+, 100); HRMS (ESI) *m/z*: $[M + H]^+$ Calcd for $C_{22}H_{22}N_2O_4H$ 379.1652; Found 379.1655.

(2*S*)-2-Amino-3-[4’-(3”-(4’”-trifluoromethylphenyl)-4”-methoxyphenyl)pyridin-2’-yl]propanoic acid hydrochloride (5b)

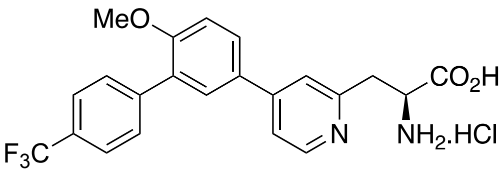

(2*S*)-2-[(Benzyloxycarbonyl)amino]-3-[4’-(3”-(4’”-trifluoromethylphenyl)-4”-methoxyphenyl)pyridin-2’-yl]propanoic acid was synthesized as described above using methyl (2*S*)-2-[(benzyloxycarbonyl)amino]-3-[4’-(3”-(4’”-trifluoromethylphenyl)-4”-methoxyphenyl)pyridin-2’-yl]propanoate (**12b**) (0.0700 g, 0.124 mmol), methanol (3 mL), 1,4-dioxane (3 mL), caesium carbonate (0.0530 g, 0.160 mmol) and water (1.5 mL). This gave (2*S*)-2-[(benzyloxycarbonyl)amino]-3-[4’-(3”-(4’”-trifluoromethylphenyl)-4”-methoxyphenyl)pyridin-2’-yl]propanoic acid as a yellow solid (0.0620 g, 91%) which was used for the next reaction without any further purification. (2*S*)-2-[(Benzyloxycarbonyl)amino]-3-[4’-(3”-(4’”-trifluoromethylphenyl)-4”-methoxyphenyl)pyridin-2’-yl]propanoic acid (0.0500 g, 0.0910 mmol) was suspended in 6 M aqueous hydrochloric acid (5 mL) and heated under reflux for 1 h. The reaction mixture was cooled to room temperature and concentrated *in vacuo*. Purification by recrystallization from a mixture of methanol and diethyl ether gave (2*S*)-2-amino-3-[4’-(3”-(4’”-trifluoromethylphenyl)-4”-methoxyphenyl)pyridin-2’-yl]propanoic acid hydrochloride (**5b**) as a yellow solid (0.0770 g, 77%). Mp 234−236 °C (decomposition); IR (neat) 3252, 2751, 1736, 1632, 1597, 1323, 1269, 1161, 1107, 1015, 814 cm^{-1}; $[\alpha]_D^{19}$ +13.5 (*c* 0.2, MeOH); ^1H NMR (400 MHz, CD_3OD) δ 8.70 (d, J = 5.9 Hz, 1H), 8.39 (br s, 1H), 8.22 (br d, J = 5.9 Hz, 1H), 8.10 (dd, J = 8.7, 2.0 Hz, 1H), 8.00 (d, J = 2.0 Hz, 1H), 7.78 (d, J = 8.4 Hz, 2H), 7.73 (d, J = 8.4 Hz, 2H), 7.37 (d, J = 8.7 Hz, 1H), 4.69 (t, J = 6.7 Hz, 1H), 3.94 (s, 3H), 3.71 (d, J = 6.7 Hz, 2H); $^{13}C\{^1H\}$ NMR (101 MHz, CD_3OD) δ 170.1, 161.2, 157.3, 152.8, 143.9, 142.8, 131.8, 131.44, 131.36, 131.0, 130.5 (q, $^2J_{C\text{-}F}$ = 32.2 Hz), 128.4, 126.0 (q, $^3J_{C\text{-}F}$ = 3.7 Hz), 125.8 (q, $^1J_{C\text{-}F}$ = 271.0 Hz), 124.5, 122.9, 113.8, 56.6, 53.0, 35.3; MS (ESI) *m/z* 417 (M + H^+, 100); HRMS (ESI) *m/z*: $[M + H]^+$ Calcd for $C_{22}H_{19}F_3N_2O_3H$ 417.1421; Found 417.1423.

(2*S*)-2-Amino-3-[4'-(3''-(naphthalen-2'''-yl)-4''-methoxyphenyl)pyridin-2'-yl]propanoic acid hydrochloride (5c)

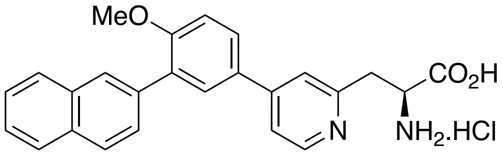

(2*S*)-2-[(Benzyloxycarbonyl)amino]-3-[4'-(3''-(naphthalen-2'''-yl)-4''-methoxyphenyl)pyridin-2'-yl]propanoic acid was synthesized as described above using methyl (2*S*)-2-[(benzyloxycarbonyl)amino]-3-[4'-(3''-(naphthalen-2'''-yl)-4''-methoxyphenyl)pyridin-2'-yl]propanoate (**12c**) (0.0800 g, 0.150 mmol), methanol (3 mL), 1,4-dioxane (3 mL), caesium carbonate (0.062 g, 0.190 mmol) and water (1.5 mL). This gave (2*S*)-2-[(benzyloxycarbonyl)amino]-3-[4'-(3''-(naphthalen-2'''-yl)-4''-methoxyphenyl)pyridin-2'-yl]propanoic acid as a yellow solid (0.0750 g, 94%) which was used for the next reaction without any further purification. (2*S*)-2-[(benzyloxycarbonyl)amino]-3-[4'-(3''-(naphthalen-2'''-yl)-4''-methoxyphenyl)pyridin-2'-yl]propanoic acid (0.0500 g, 0.0940 mmol) was suspended in 6 M aqueous hydrochloric acid (5 mL) and heated under reflux for 1 h. The reaction mixture was cooled to room temperature and concentrated *in vacuo*. Purification by recrystallization from a mixture of methanol and diethyl ether gave (2*S*)-2-amino-3-[4'-(3''-(naphthalen-2'''-yl)-4''-methoxyphenyl)pyridin-2'-yl]propanoic acid hydrochloride (**5c**) as a yellow solid (0.0337 g, 82%). Mp 110−115 °C (decomposition); IR (neat) 3372, 2924, 1732, 1632, 1593, 1481, 1265, 1242, 1153, 1015, 814 cm^{-1}; $[\alpha]_D^{19}$ –4.8 (*c* 0.2, MeOH); ^1H NMR (400 MHz, CD_3OD) δ 8.68 (d, *J* = 5.8 Hz, 1H), 8.31 (br s, 1H), 8.18 (br d, *J* = 5.8 Hz, 1H), 8.09−7.99 (m, 3H), 7.95−7.86 (m, 3H), 7.70 (dd, *J* = 8.5, 1.4 Hz, 1H), 7.55−7.47 (m, 2H), 7.36 (d, *J* = 8.4 Hz, 1H), 4.68 (t, *J* = 6.7 Hz, 1H), 3.95 (s, 3H), 3.67 (d, *J* = 6.7 Hz, 2H); $^{13}C\{^1H\}$ NMR (101 MHz, CD_3OD) δ 170.1, 161.6, 157.7, 152.6, 143.7, 136.4, 134.8, 134.2, 133.5, 131.7, 130.4, 129.4, 129.2, 128.9, 128.6, 128.4, 128.2, 127.24, 127.20, 124.9, 122.9, 113.6, 56.6, 53.0, 35.3; MS (ESI) *m/z* 399 (M + H^+, 100); HRMS (ESI) *m/z*: $[M + H]^+$ Calcd for $C_{25}H_{22}N_2O_3H$ 399.1703; Found 399.1706.

3. Computational Details

All DFT calculations were performed in Gaussian 16[6] with the ωB97X-D functional[7] and the def2-TZVP basis set.[8] The methanol solvent was included using the default PCM solvent model. The structure of amino acid **5a** was fully optimized (no imaginary frequencies), and regular population analysis was performed. Visualizations of the HOMO and LUMO were generated using Avogadro software.[9]

Optimized structure and total energy of **5a**:

```
50
5a E = -1262.07362301
N     -6.40177   -1.42426    0.77342
C     -6.15130   -0.10552    0.12249
C     -4.94704   -0.20044   -0.81601
C     -3.66306   -0.58813   -0.13094
C     -2.53880    0.21981   -0.17796
C     -1.36347   -0.18691    0.44804
C     -1.38424   -1.41244    1.10901
C     -2.55020   -2.15296    1.11749
N     -3.66715   -1.76147    0.51119
C     -7.42974    0.25751   -0.68675
O     -7.50053    1.42424   -1.09151
O     -8.22670   -0.69149   -0.86237
H     -5.52828   -1.98175    0.79092
H     -6.75162   -1.32166    1.72094
H     -5.97258    0.63606    0.89785
H     -4.84087    0.76723   -1.30184
H     -5.16621   -0.93539   -1.59655
H     -2.57741    1.15474   -0.72125
H     -0.51261   -1.78040    1.63264
H     -2.59253   -3.10273    1.63806
C     -0.14634    0.65275    0.41212
C      1.11197    0.06986    0.29865
H      1.19511   -1.00810    0.22946
C      2.28153    0.82320    0.26647
C      2.16264    2.21555    0.35244
O      3.26469    3.02640    0.34545
C      0.91528    2.81027    0.47236
```

H	0.86025	3.88896	0.54294
C	-0.23219	2.03943	0.50231
H	-1.19529	2.52057	0.61587
C	3.83199	3.24029	-0.94063
H	3.11022	3.73015	-1.60042
H	4.15694	2.29915	-1.39172
H	4.69389	3.88849	-0.79867
H	2.98545	-1.08049	-1.50967
C	3.79006	-0.83417	-0.82719
C	5.00237	-1.49963	-0.95780
H	5.11203	-2.24854	-1.72859
C	6.05152	-1.18635	-0.10136
O	7.27104	-1.76861	-0.13934
C	5.86772	-0.20825	0.87691
H	6.68783	0.02413	1.54415
C	4.65913	0.44550	0.99053
H	4.53566	1.20067	1.75509
C	3.59350	0.14421	0.13863
C	7.50675	-2.76818	-1.11206
H	8.53193	-3.09781	-0.96448
H	7.39710	-2.37065	-2.12454
H	6.83128	-3.61761	-0.98029
H	-7.15442	-1.84150	0.19954

4. References

1. Fowler, L. S.; Ellis, D.; Sutherland, A. Synthesis of Fluorescent Enone Derived α-Amino Acids. *Org. Biomol. Chem.* **2009**, *7*, 4309–4316.

2. Williams, A. T. R.; Winfield, S. A.; Miller, J. N. Relative Fluorescence Quantum Yields Using a Computer-controlled Luminescence Spectrometer. *Analyst* **1983**, *108*, 1067–1071.

3. Rudisill, D. E.; Whitten, J. P. Synthesis of (*R*)-4-Oxo-5-Phosphononorvaline, an *N*-Methyl-D-Aspartic Acid Receptor Selective β-Keto Phosphonate. *Synthesis* **1994**, 851–854.

4. Gilfillan, L.; Artschwager, R.; Harkiss, A. H.; Liskamp, R. M. J.; Sutherland, A. Synthesis of Pyrazole Containing α-Amino Acids via a Highly Regioselective Condensation/Aza-Michael Reaction of β-Aryl α,β-Unsaturated Ketones. *Org. Biomol. Chem.* **2015**, *13*, 4514–4523.

5. Harkiss, A. H.; Bell, J. D.; Knuhtsen, A.; Jamieson, A. G.; Sutherland, A. Synthesis and Fluorescent Properties of β-Pyridyl α-Amino Acids. *J. Org. Chem.* **2019**, *84*, 2879–2890.

6. Frisch, M. J.; Trucks, G. W.; Schlegel, H. B.; Scuseria, G. E.; Robb, M. A.; Cheeseman, J. R.; Scalmani, G.; Barone, V.; Petersson, G. A.; Nakatsuji, H.; Li, X.; Caricato, M.; Marenich, A. V.; Bloino, J.; Janesko, B. G.; Gomperts, R.; Mennucci, B.; Hratchian, H. P.; Ortiz, J. V.; Izmaylov, A. F.; Sonnenberg, J. L.; Williams-Young, D.; Ding, F.; Lipparini, F.; Egidi, F.; Goings, J.; Peng, B.; Petrone, A.; Henderson, T.; Ranasinghe, D.; Zakrzewski, V. G.; Gao, J.; Rega, N.; Zheng, G.; Liang, W.; Hada, M.; Ehara, M.; Toyota, K.; Fukuda, R.; Hasegawa, J.; Ishida, M.; Nakajima, T.; Honda, Y.; Kitao, O.; Nakai, H.; Vreven, T.; Throssell, K.; Montgomery, Jr, J. A.; Peralta, J. E.; Ogliaro, F.; Bearpark, M. J.; Heyd, J. J.; Brothers, E. N.; Kudin, K. N.; Staroverov, V. N.; Keith, T. A.; Kobayashi, R.; Normand, J.; Raghavachari, K.; Rendell, A. P.; Burant, J. C.; Iyengar, S. S.; Tomasi, J.; Cossi, M.; Millam, J. M.; Klene, M.; Adamo, C.; Cammi, R.; Ochterski, J. W.; Martin, R. L.; Morokuma, K.; Farkas, O.; Foresman, J. B.; Fox, D. J. Gaussian 16, Gaussian, Inc., Wallingford CT, Revision C.01, **2016**.

7. Chai, J.-D.; Head-Gordon, M. *Phys. Chem. Chem. Phys.* **2008**, *10*, 6615–6620.

8. Weigend, F.; Ahlrichs, R.; *Phys. Chem. Chem. Phys.* **2005**, *7*, 3297–3305.

9. Avogadro: an open-source molecular builder and visualization tool. Version 1.2. http://avogadro.cc/

5. Photophysical Data for α-Amino Acids

Spectra were recorded at 5 μM in MeOH using an excitation and emission bandpass of 3 or 5 nm.

Absorption and Emission Spectra for 5a.

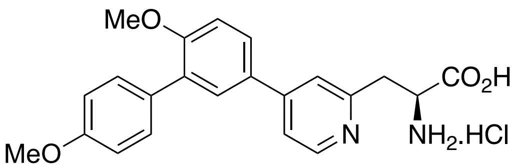

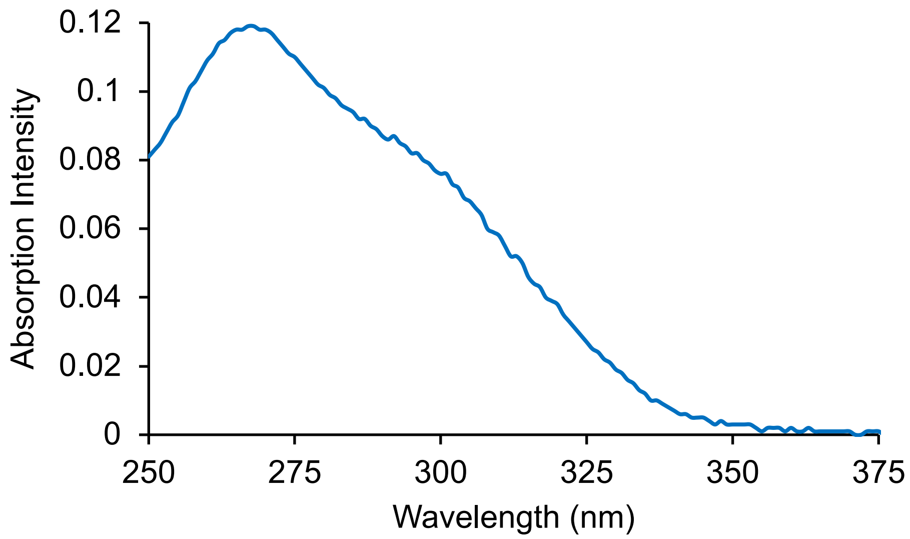

Excitation at 264 nm:

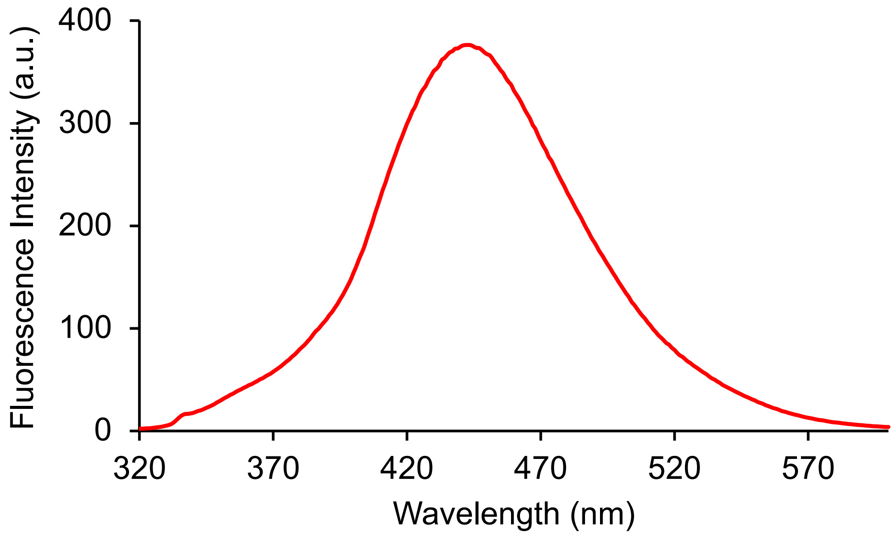

Absorption and Emission Spectra for 5b.

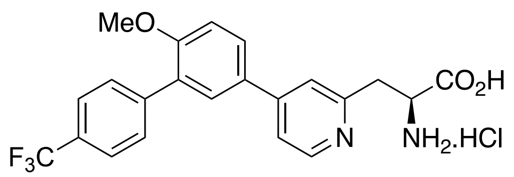

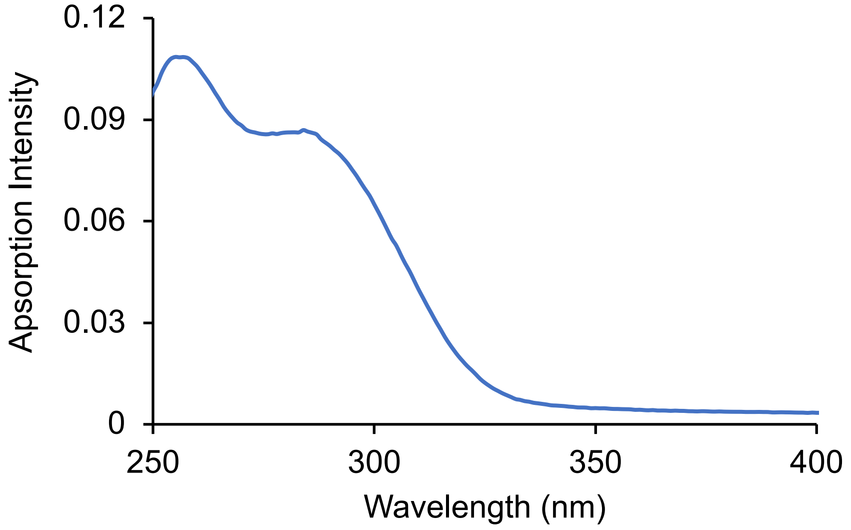

Excitation at 260 nm:

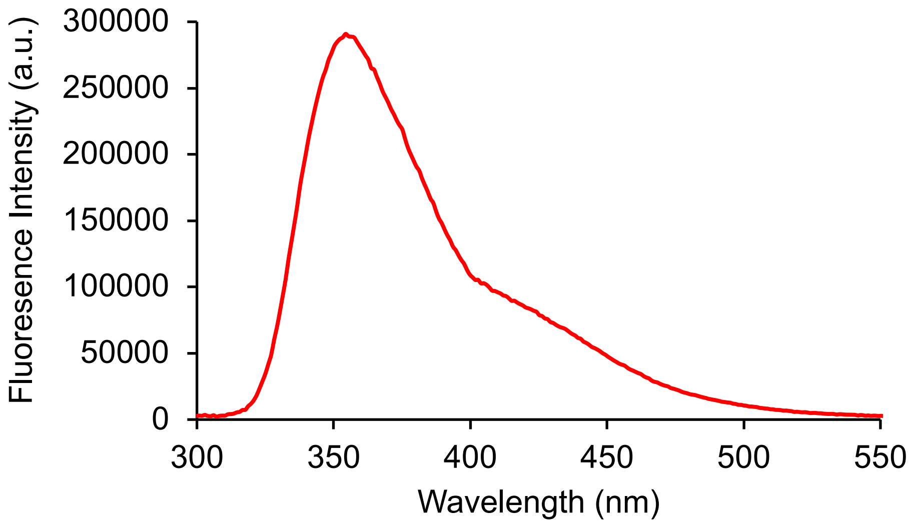

Absorption and Emission Spectra for 5c.

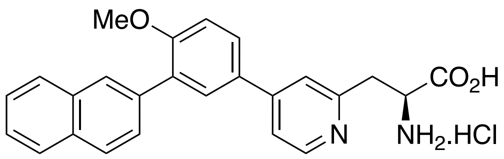

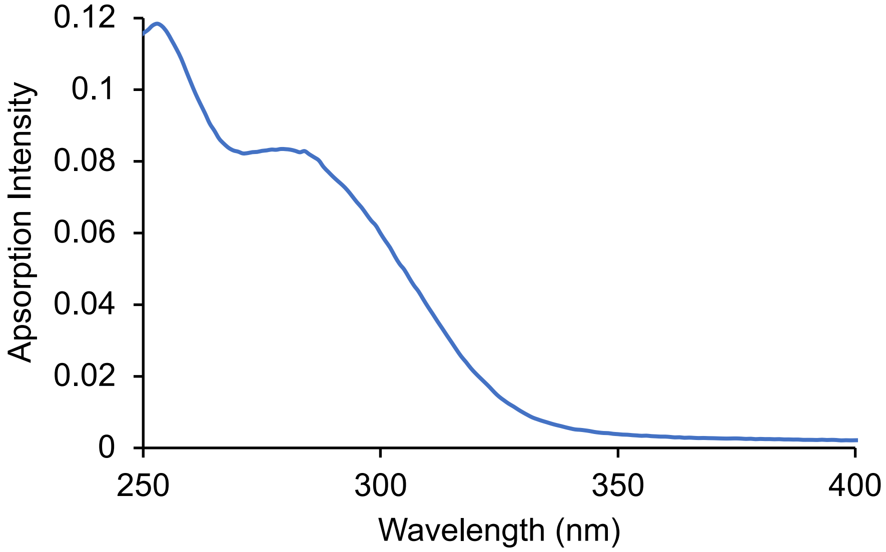

Excitation at 255 nm:

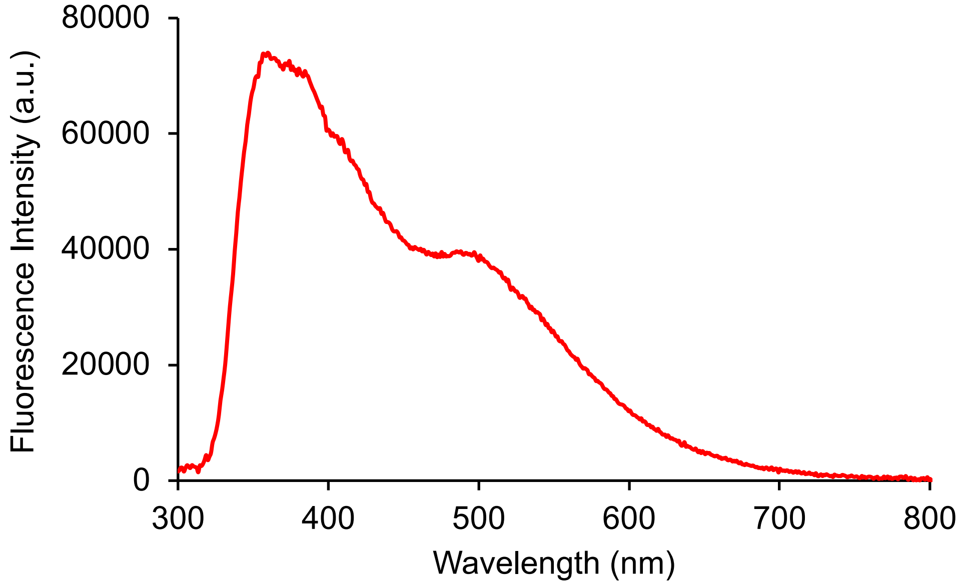

Solvatochromism with Amino Acid 5a:

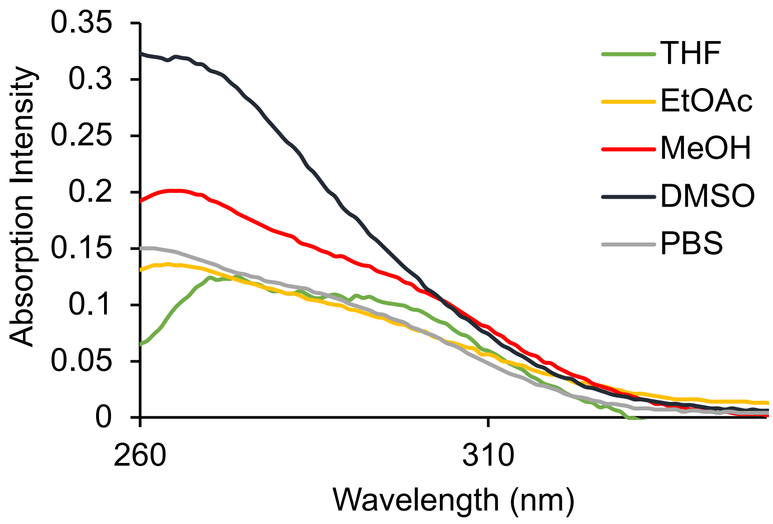

Excitation at 264 nm:

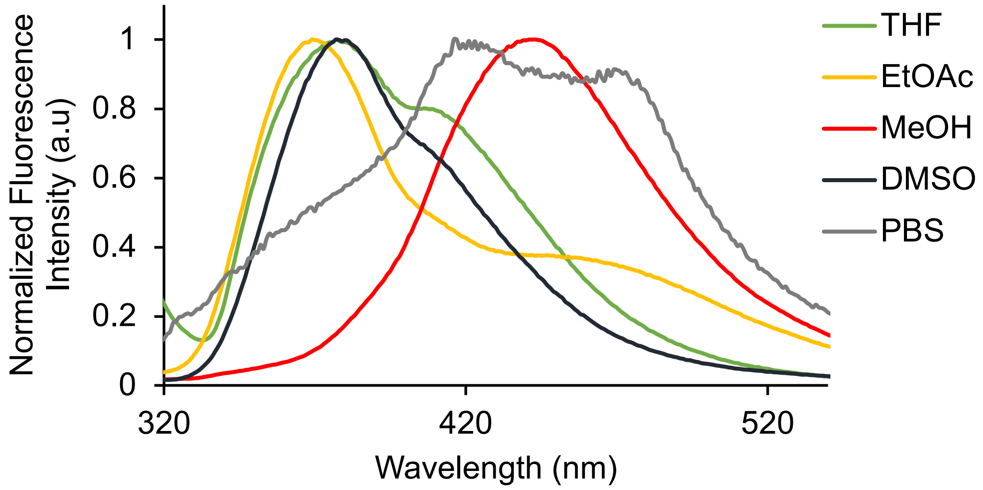

Lippert-Mataga Plot for 5a:

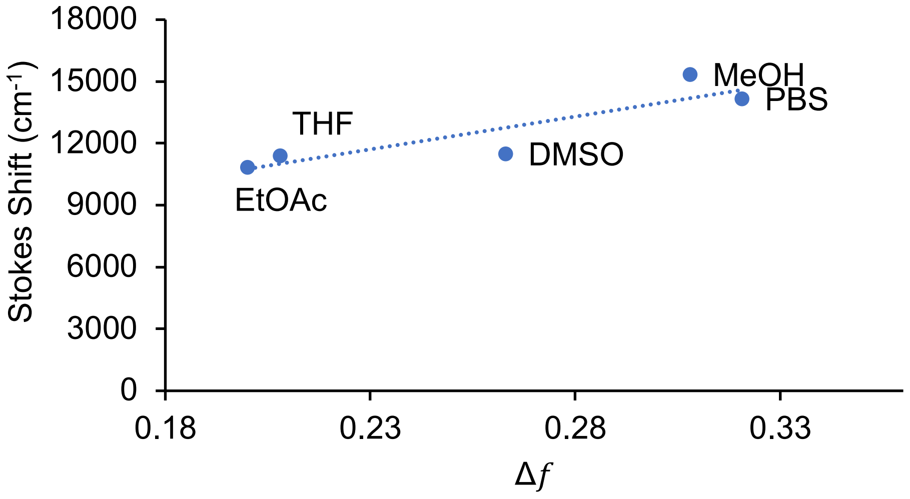

Viscosity Studies with Amino Acid 5a (in MeOH):

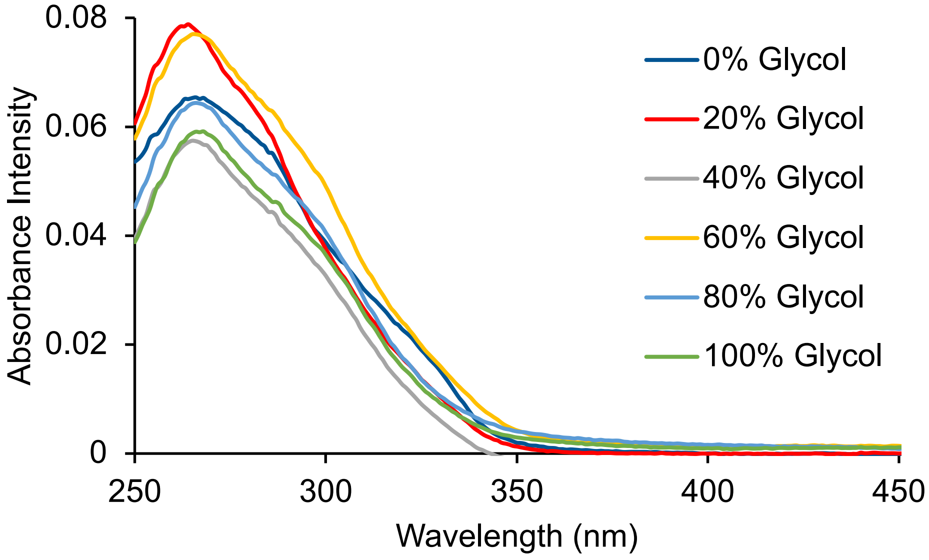

Excitation at 264 nm:

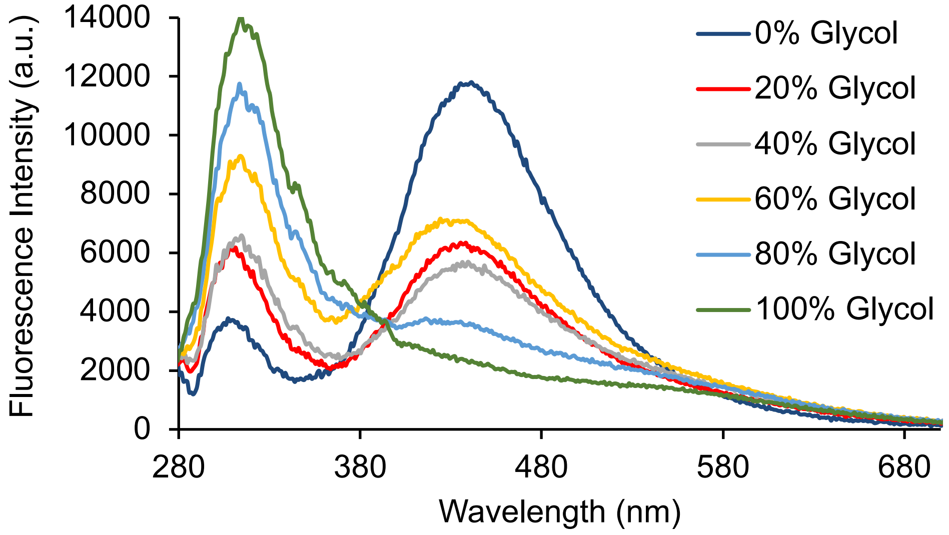

Aggregation Studies with Amino Acid 5a (in MeOH):

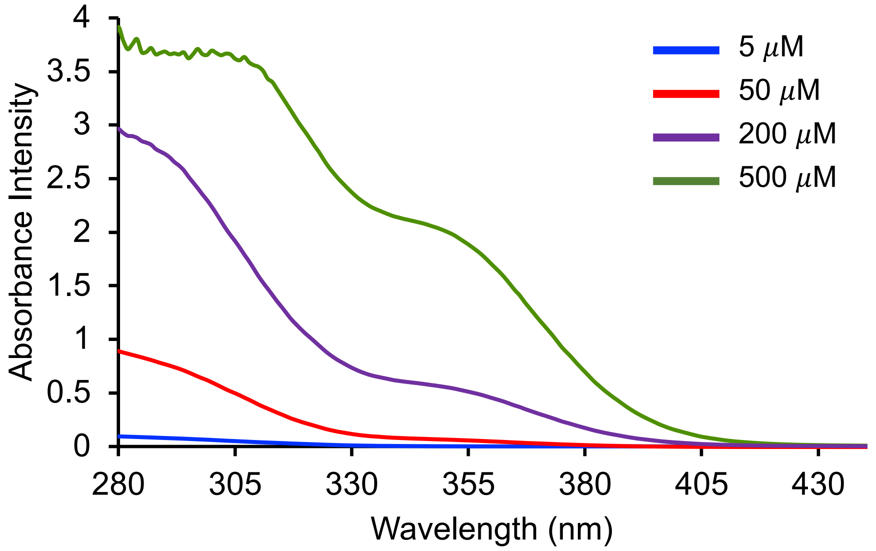

Excitation at 264 nm:

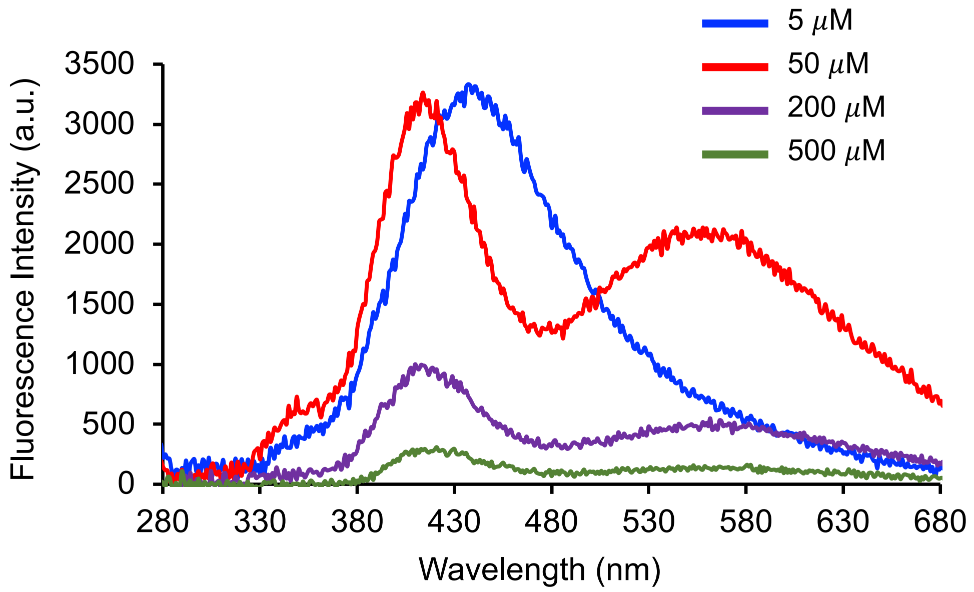

6. ^{1}H and ^{13}C NMR Spectra for all Compounds

^{1}H NMR (400 MHz, $CDCl_3$)

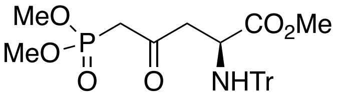

7

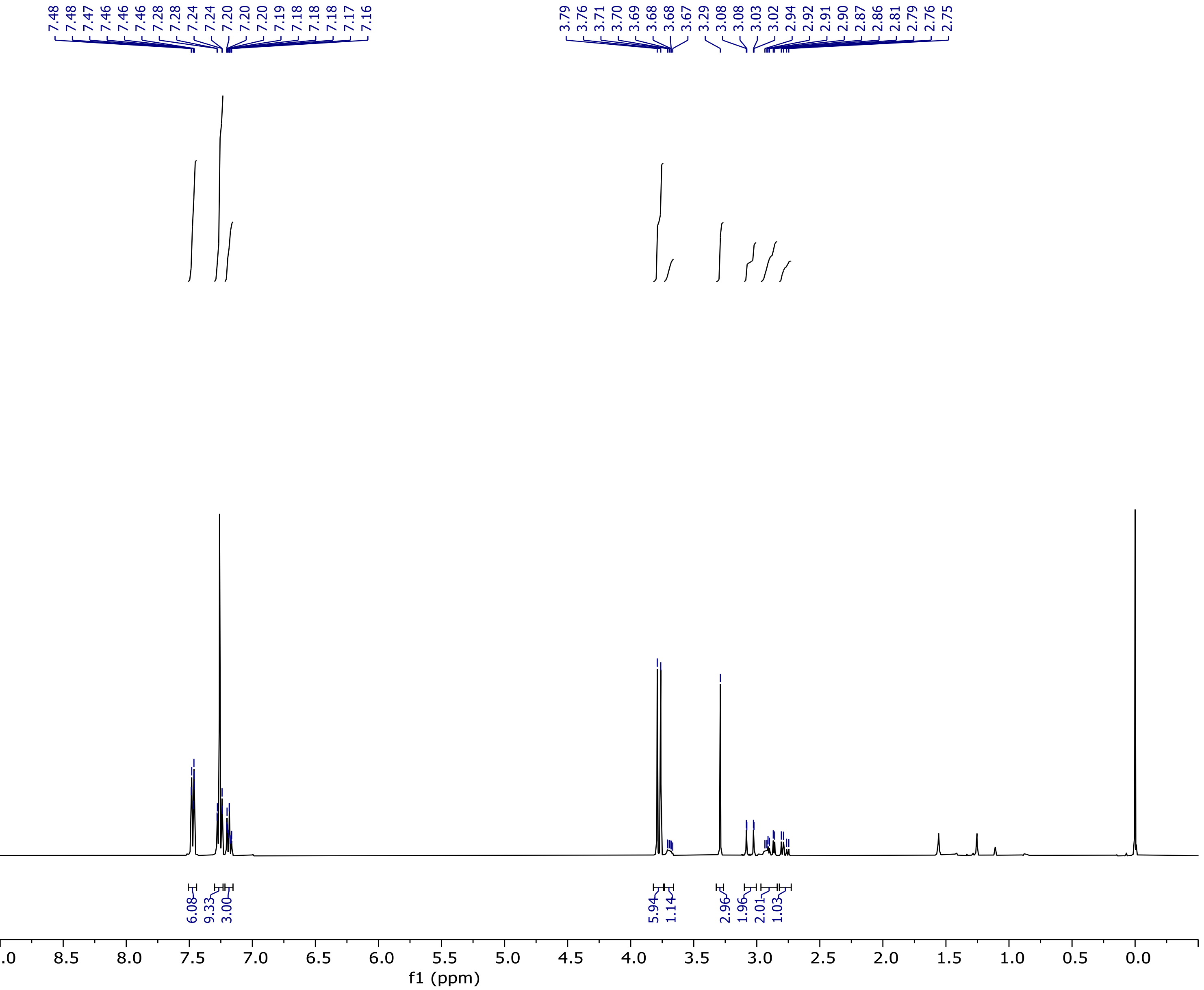

$^{13}C\{^{1}H\}$ NMR (101 MHz, $CDCl_3$)

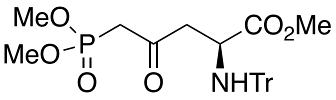

7

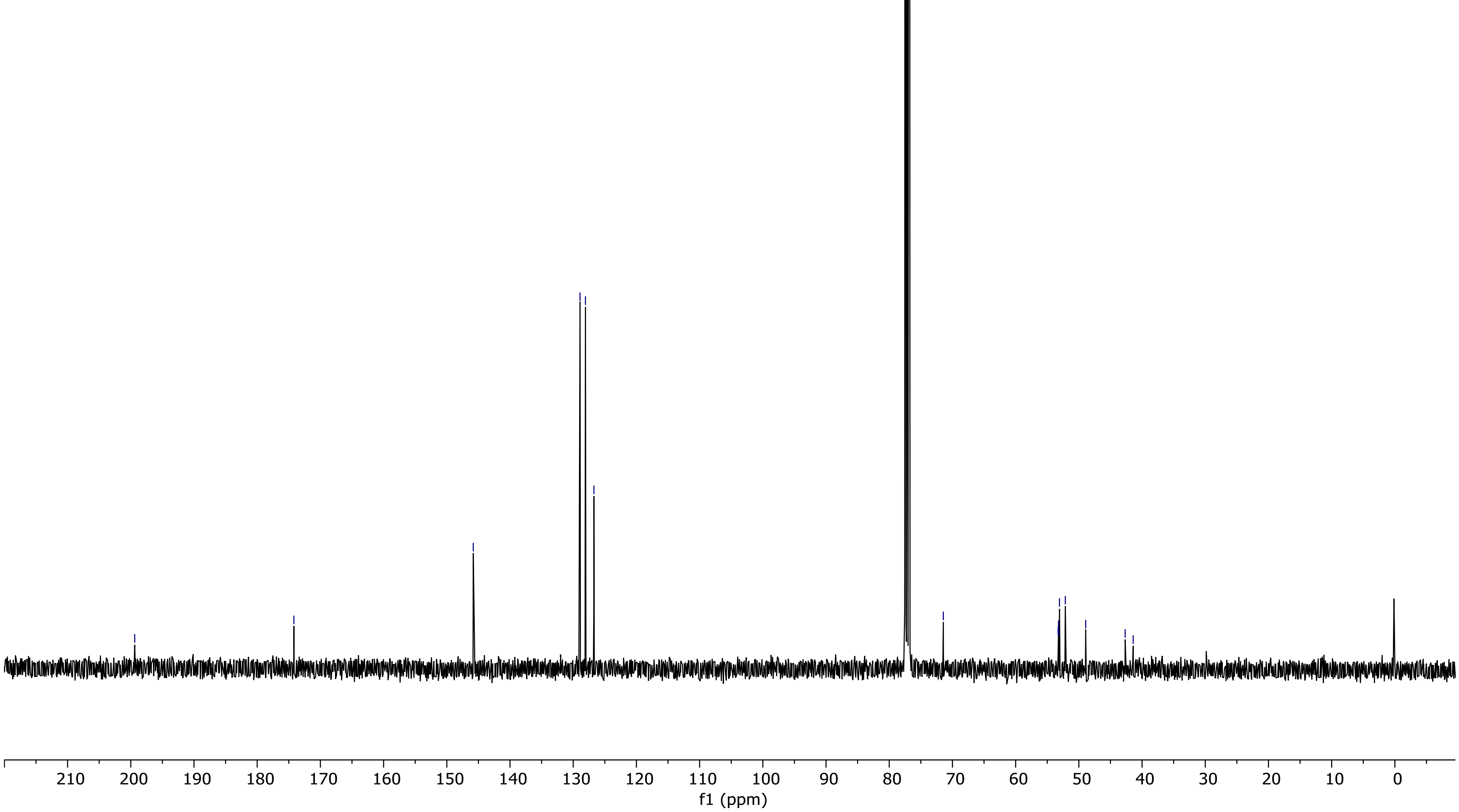

^{1}H NMR (400 MHz, $CDCl_3$)

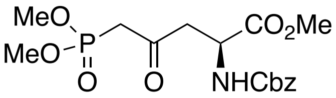

8

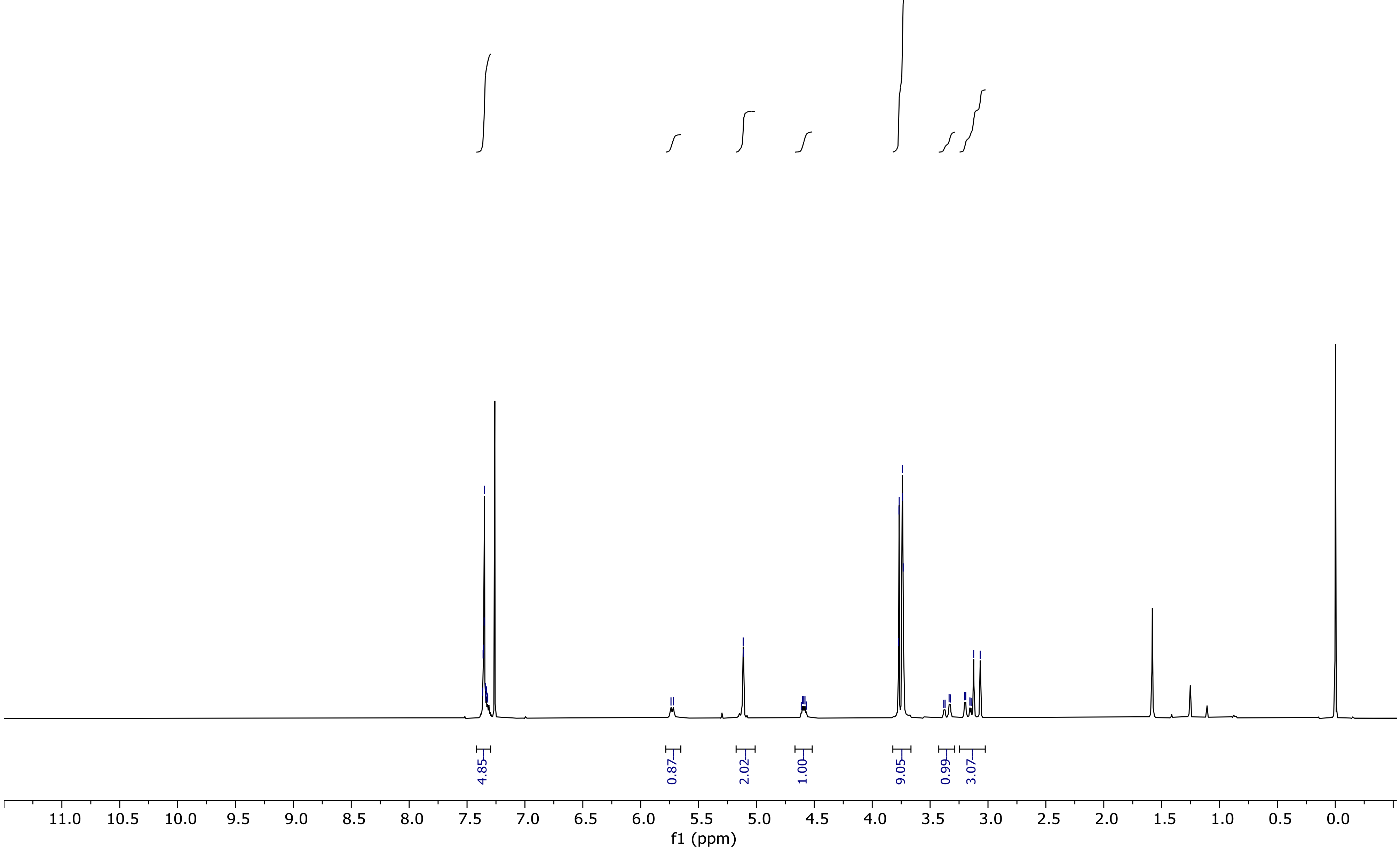

$^{13}C\{^1H\}$ NMR (101 MHz, $CDCl_3$)

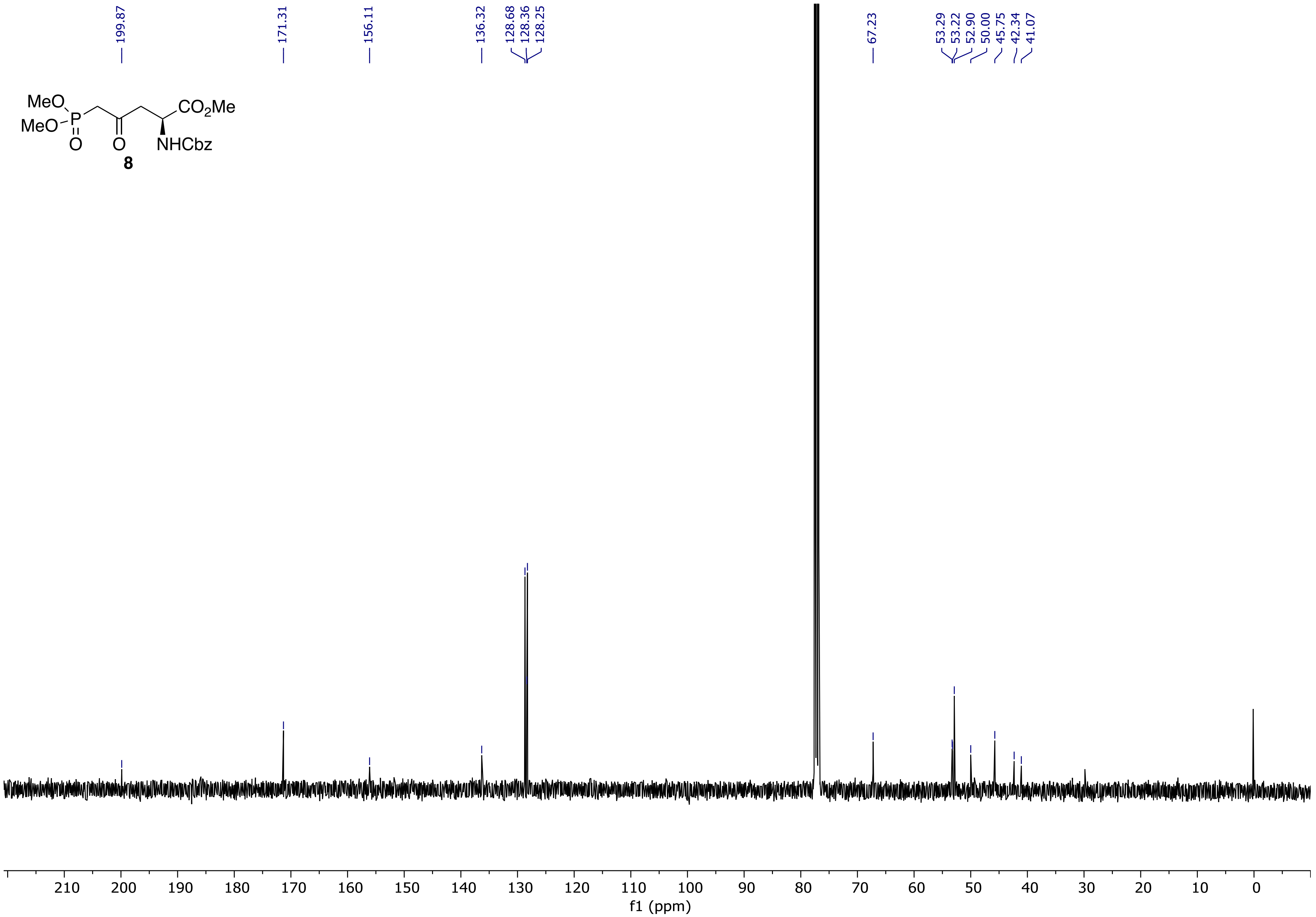

^{1}H NMR (400 MHz, $CDCl_3$)

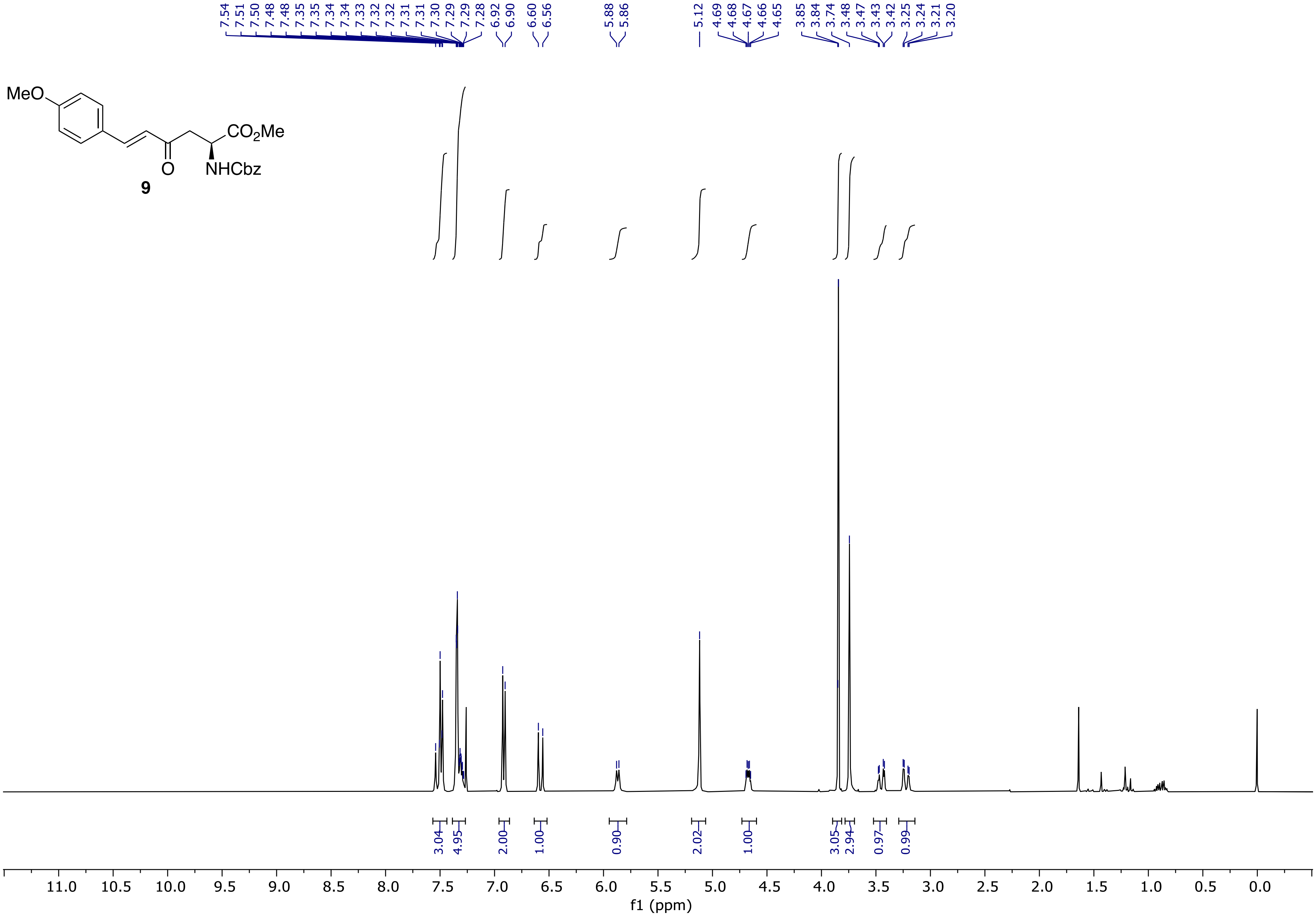

$^{13}C\{^{1}H\}$ NMR (101 MHz, $CDCl_3$)

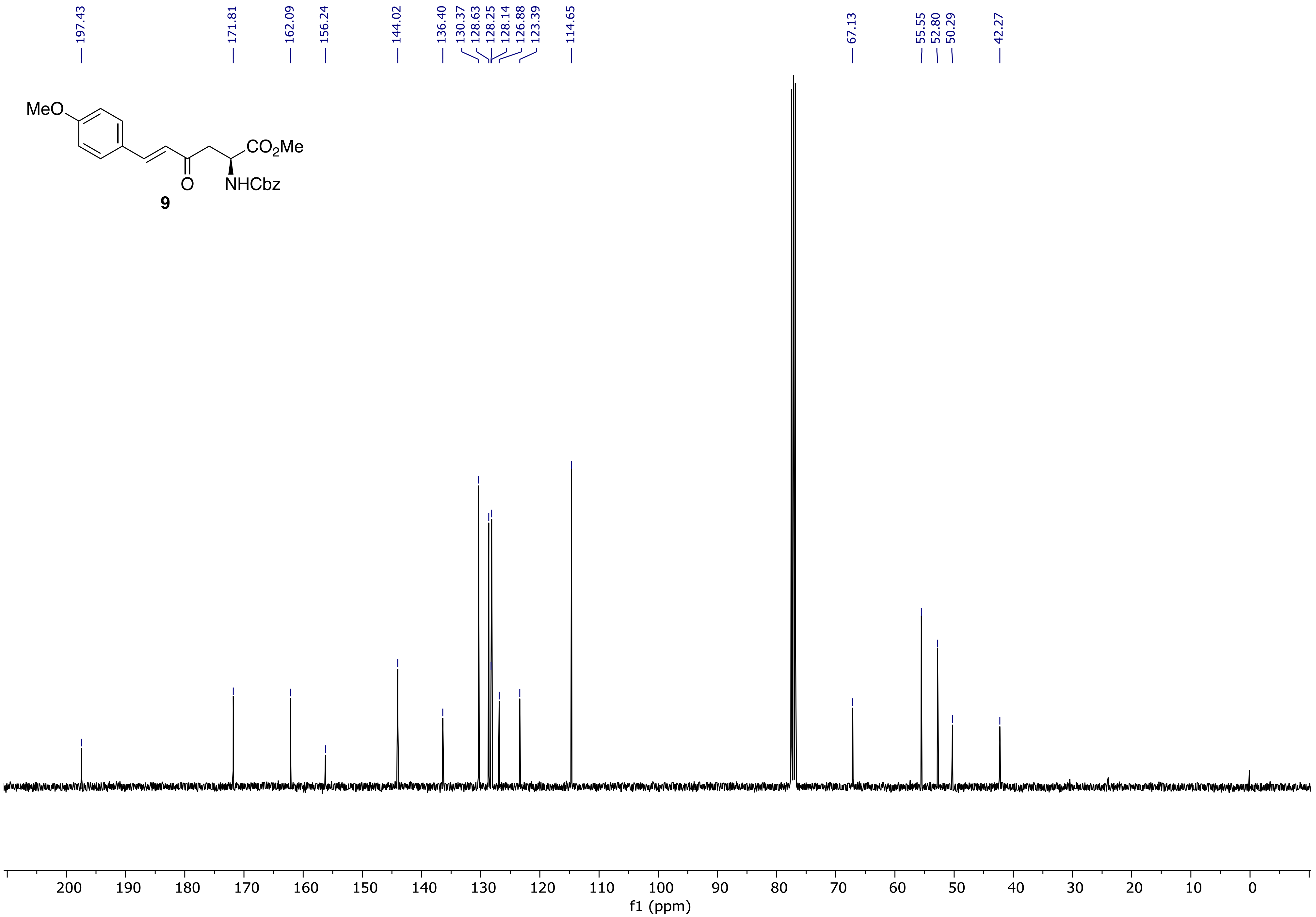

^{1}H NMR (500 MHz, CDCl$_3$)

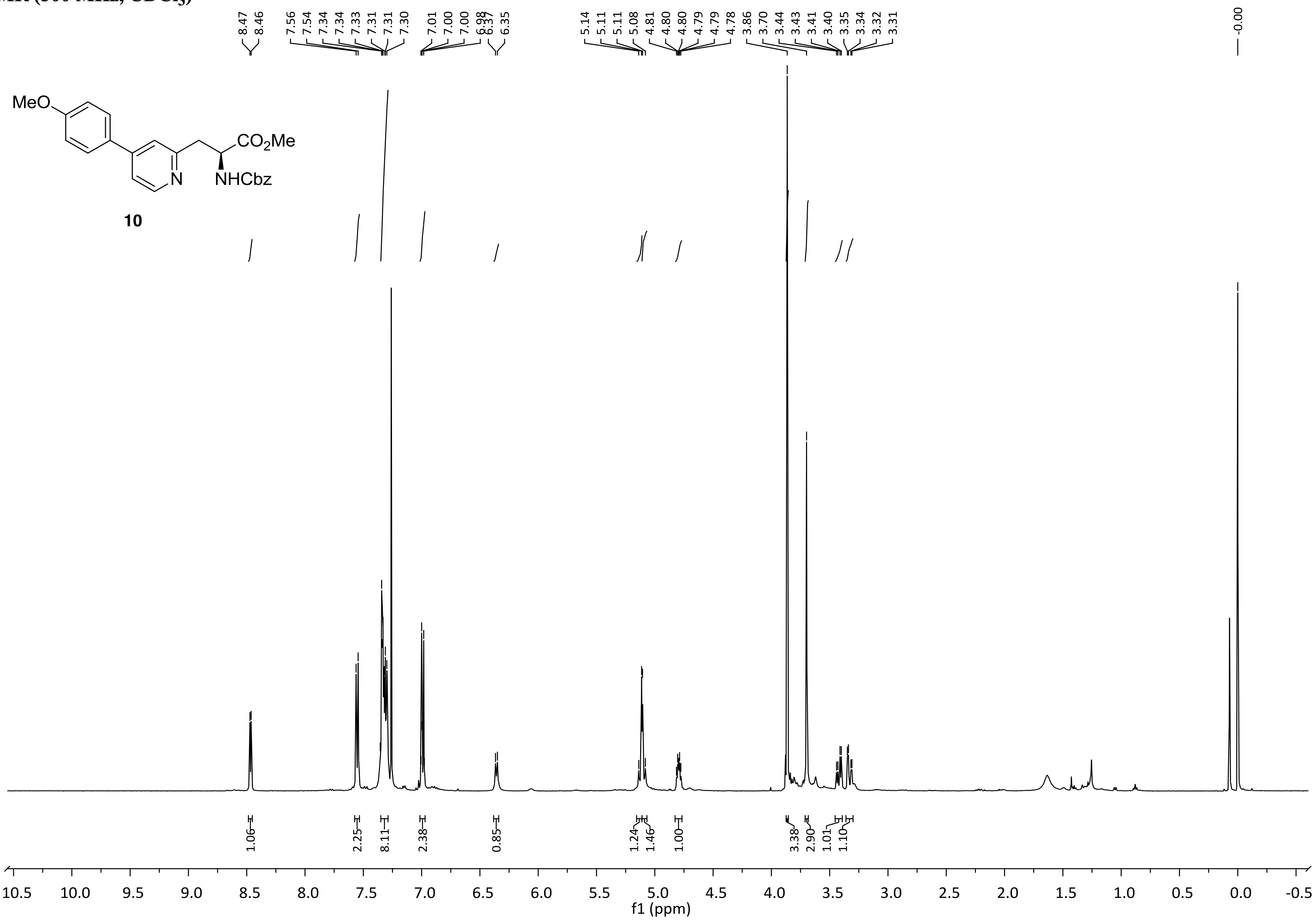

10

$^{13}C\{^1H\}$ NMR (126 MHz, $CDCl_3$)

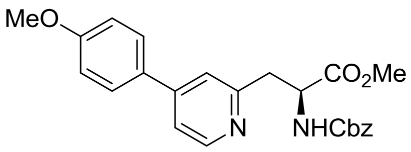

10

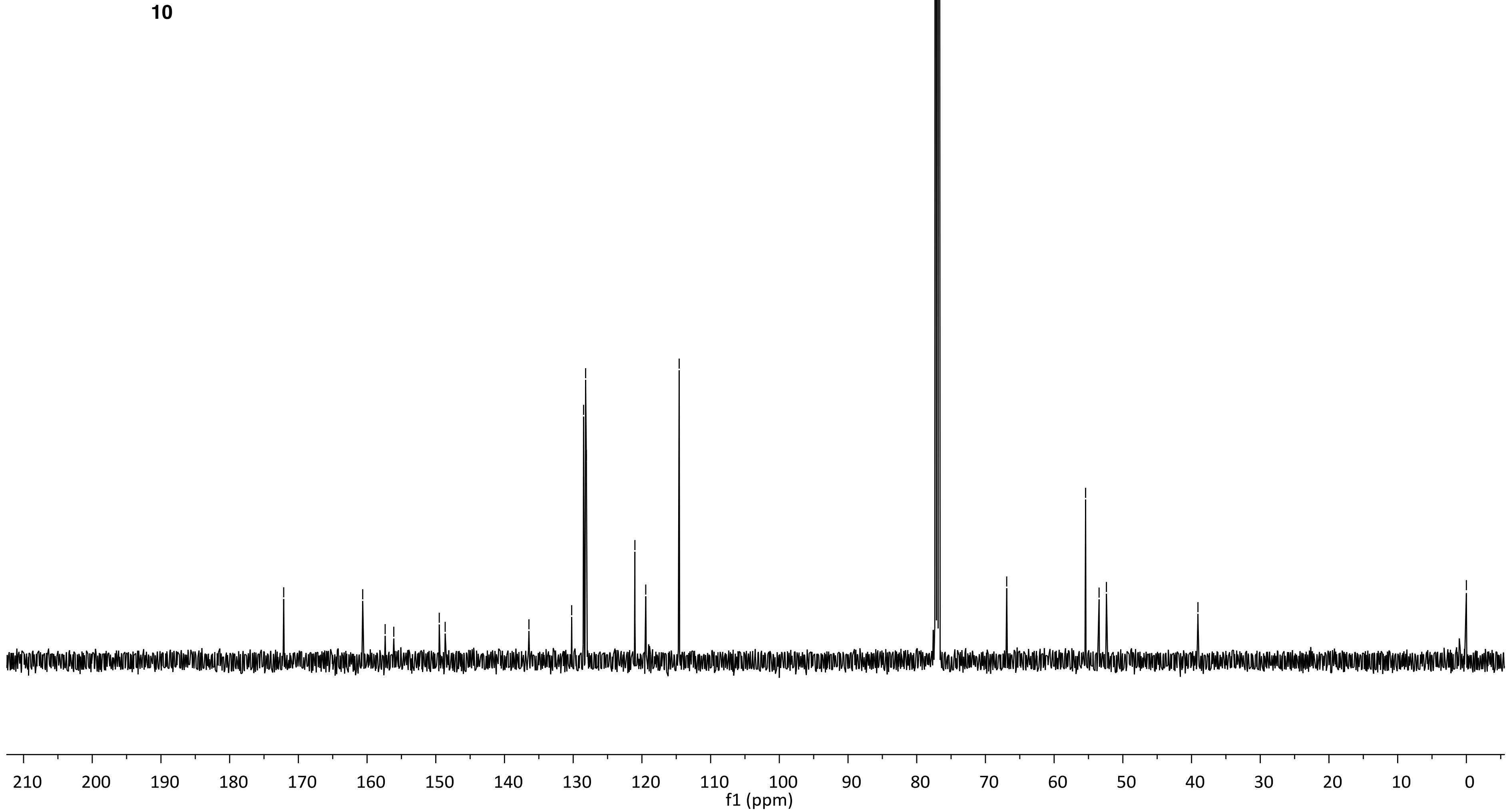

^{1}H NMR (500 MHz, $CDCl_3$)

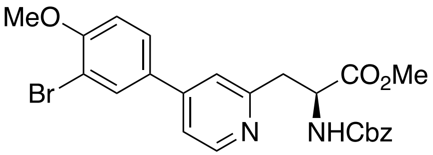

11

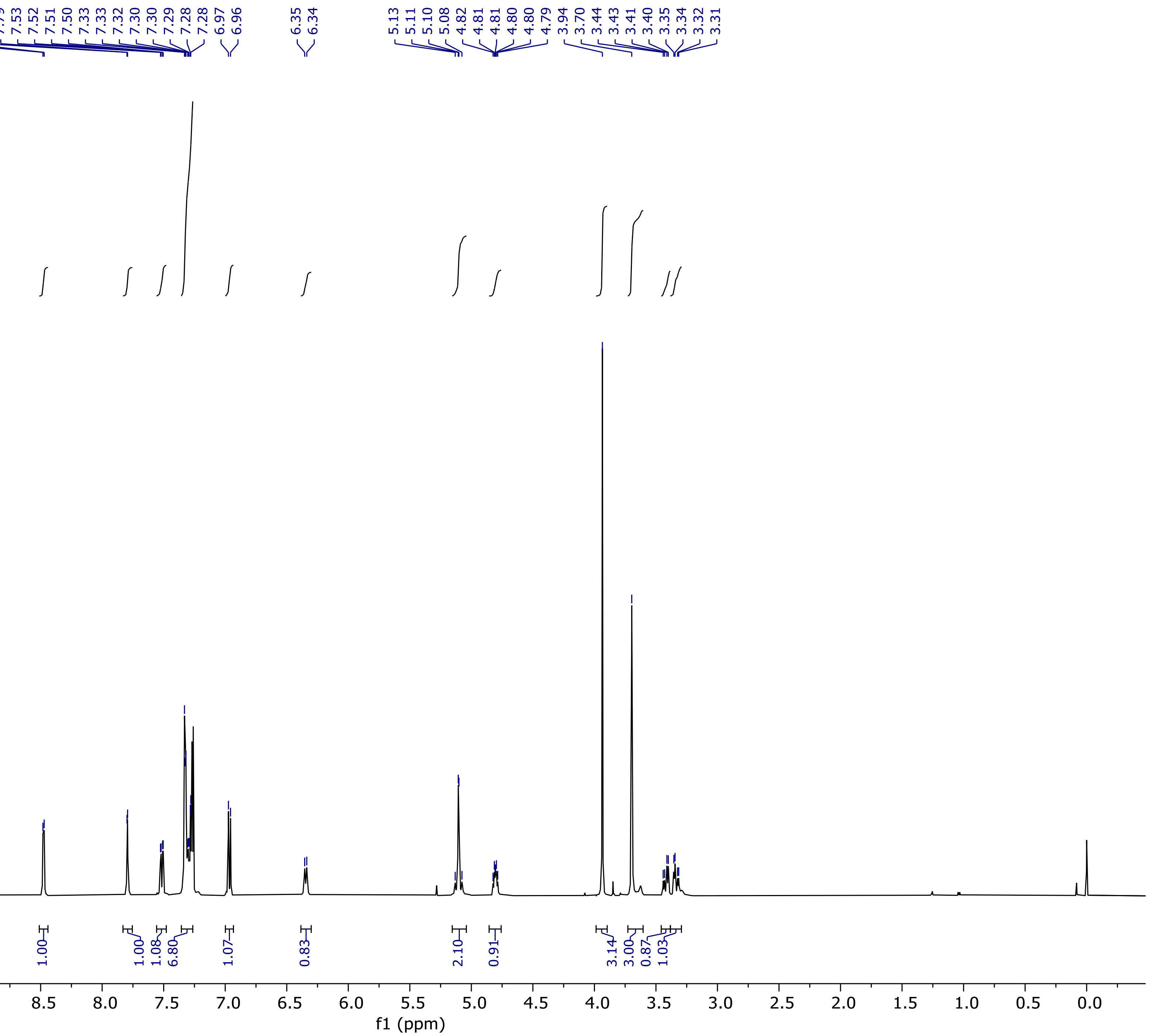

$^{13}C\{^1H\}$ NMR (126 MHz, $CDCl_3$)

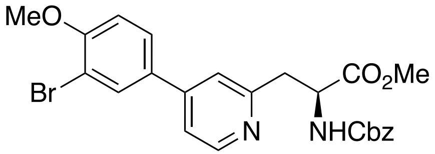

11

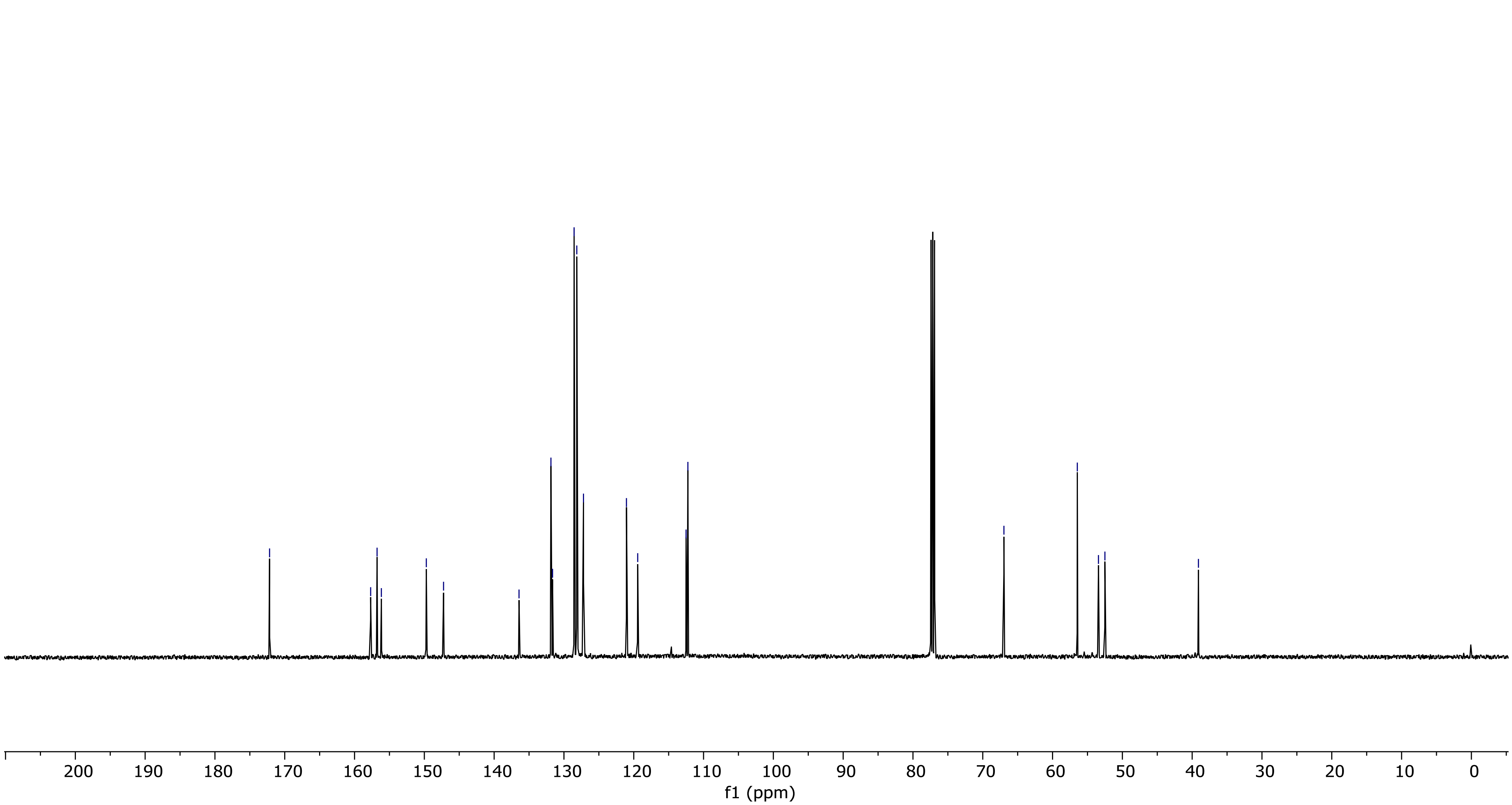

^{1}H NMR (500 MHz, $CDCl_3$)

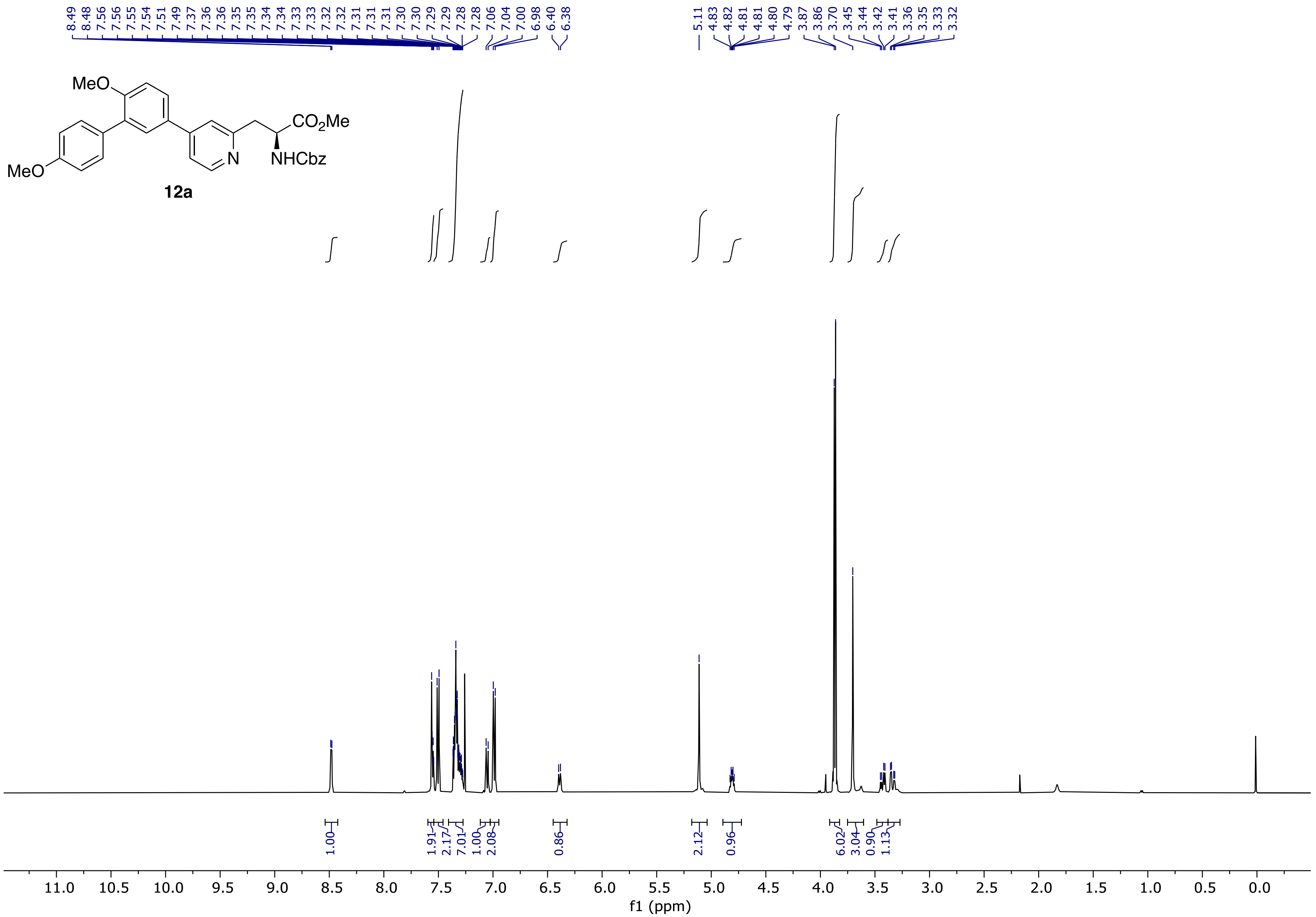

12a

$^{13}C\{^1H\}$ NMR (126 MHz, $CDCl_3$)

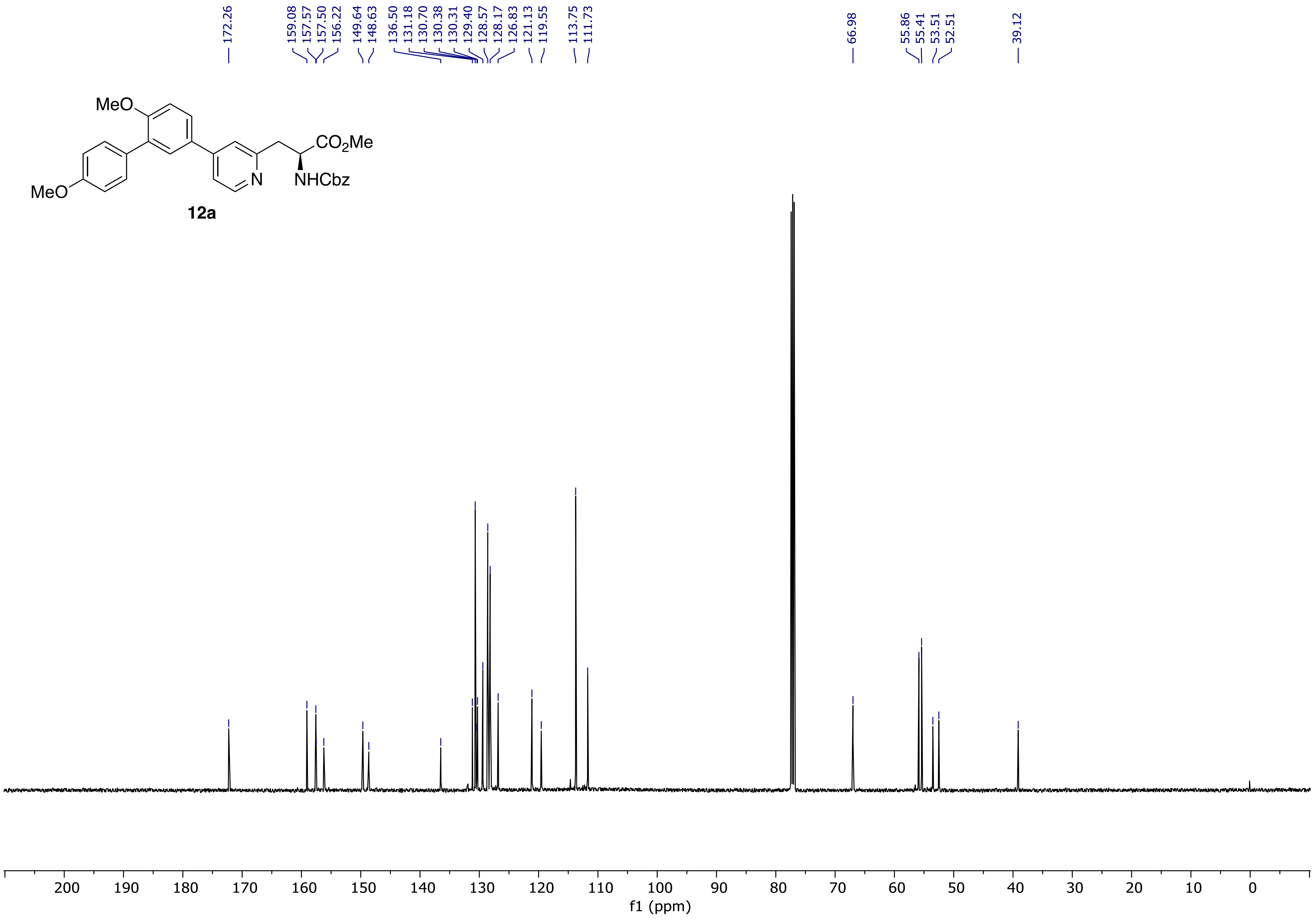

^{1}H NMR (400 MHz, $CDCl_3$)

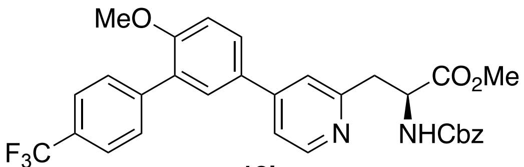

12b

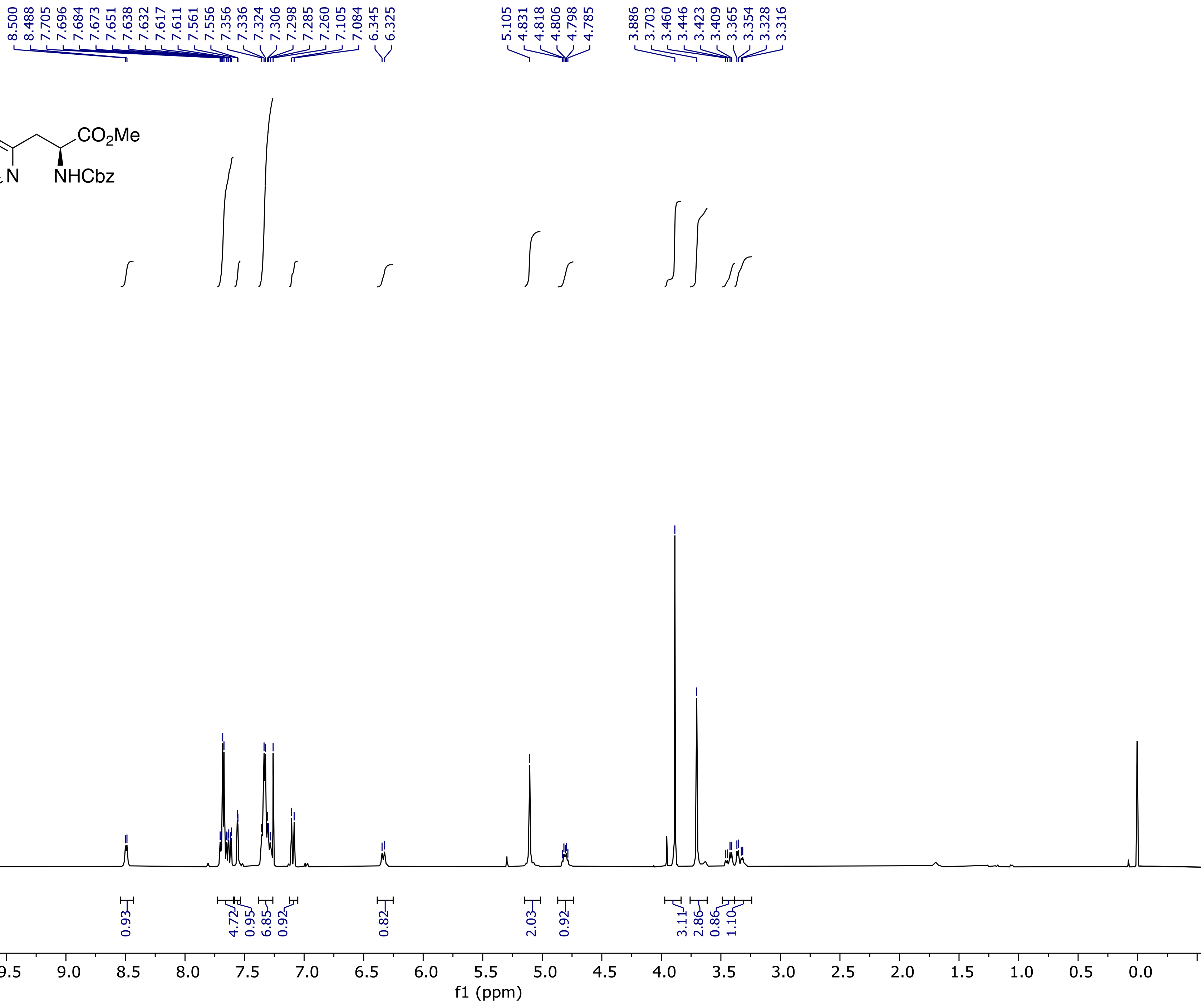

$^{13}C\{^1H\}$ NMR (101 MHz, $CDCl_3$)

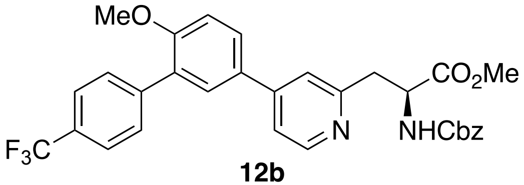

12b

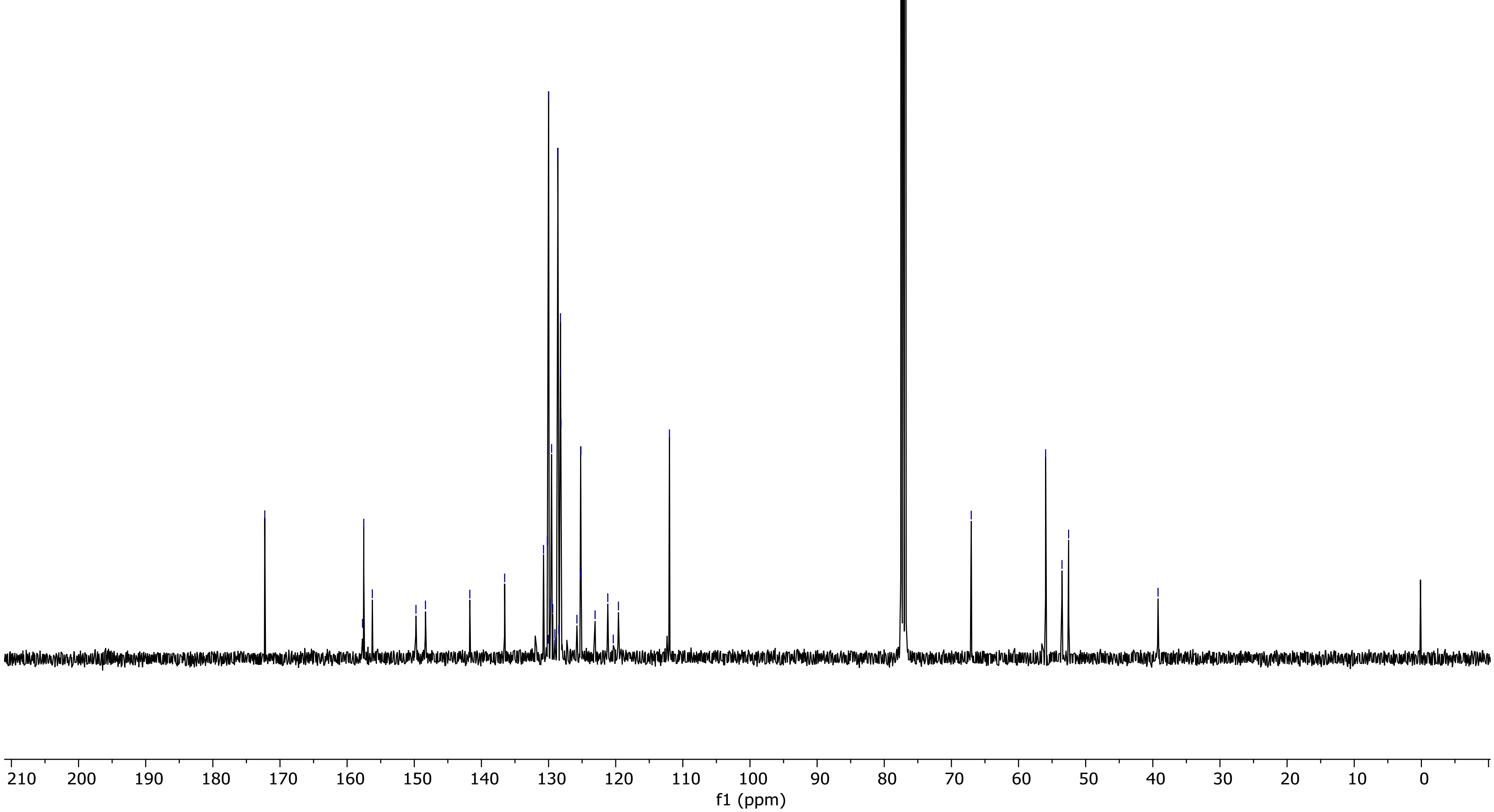

^{1}H NMR (400 MHz, $CDCl_3$)

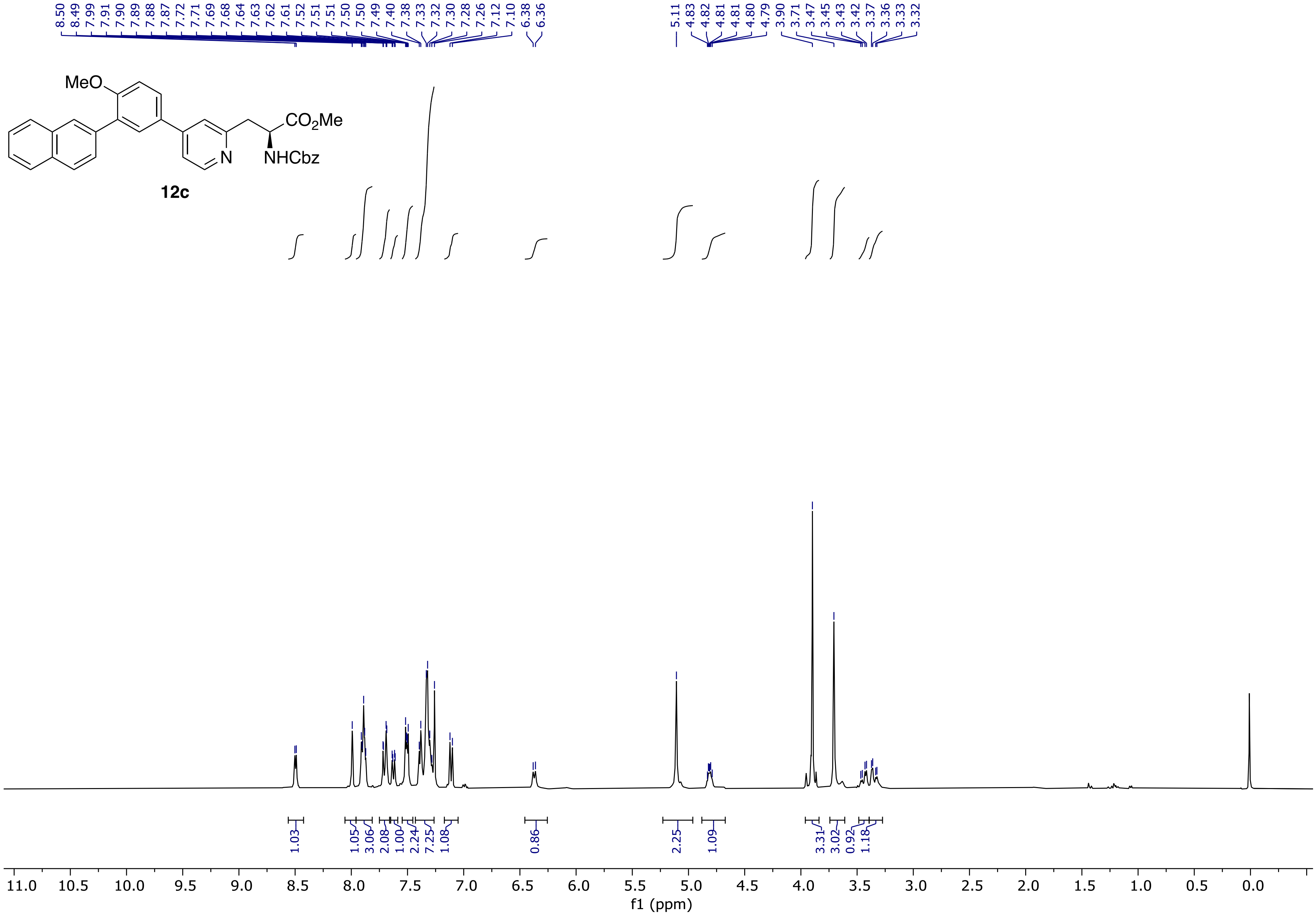

$^{13}C\{^{1}H\}$ NMR (101 MHz, $CDCl_3$)

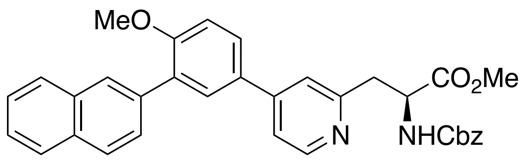

12c

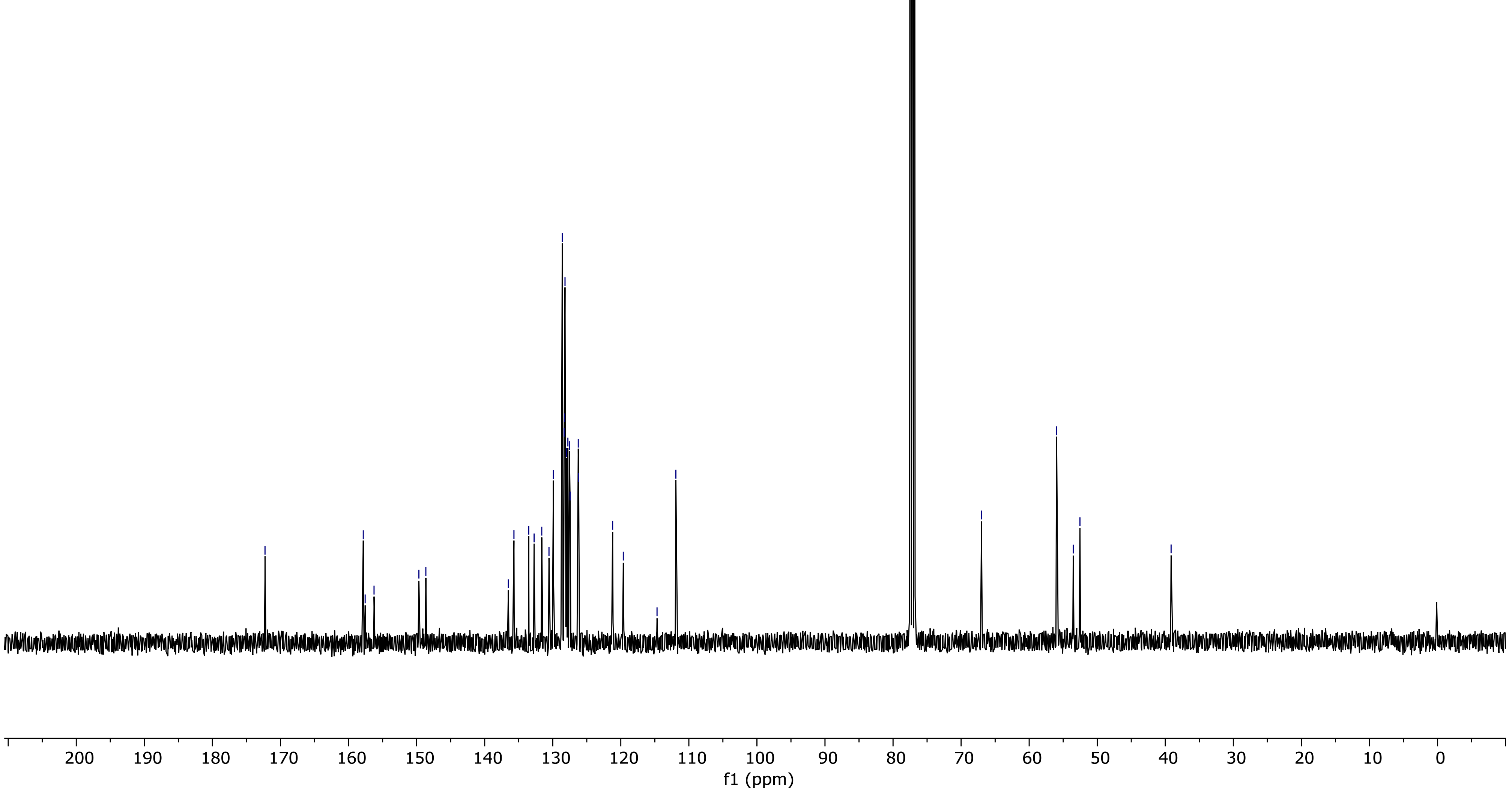

^{1}H NMR (400 MHz, CD_3OD)

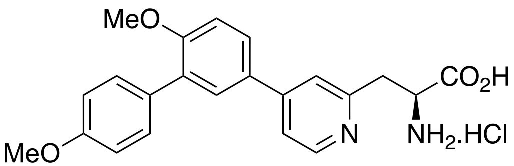

5a

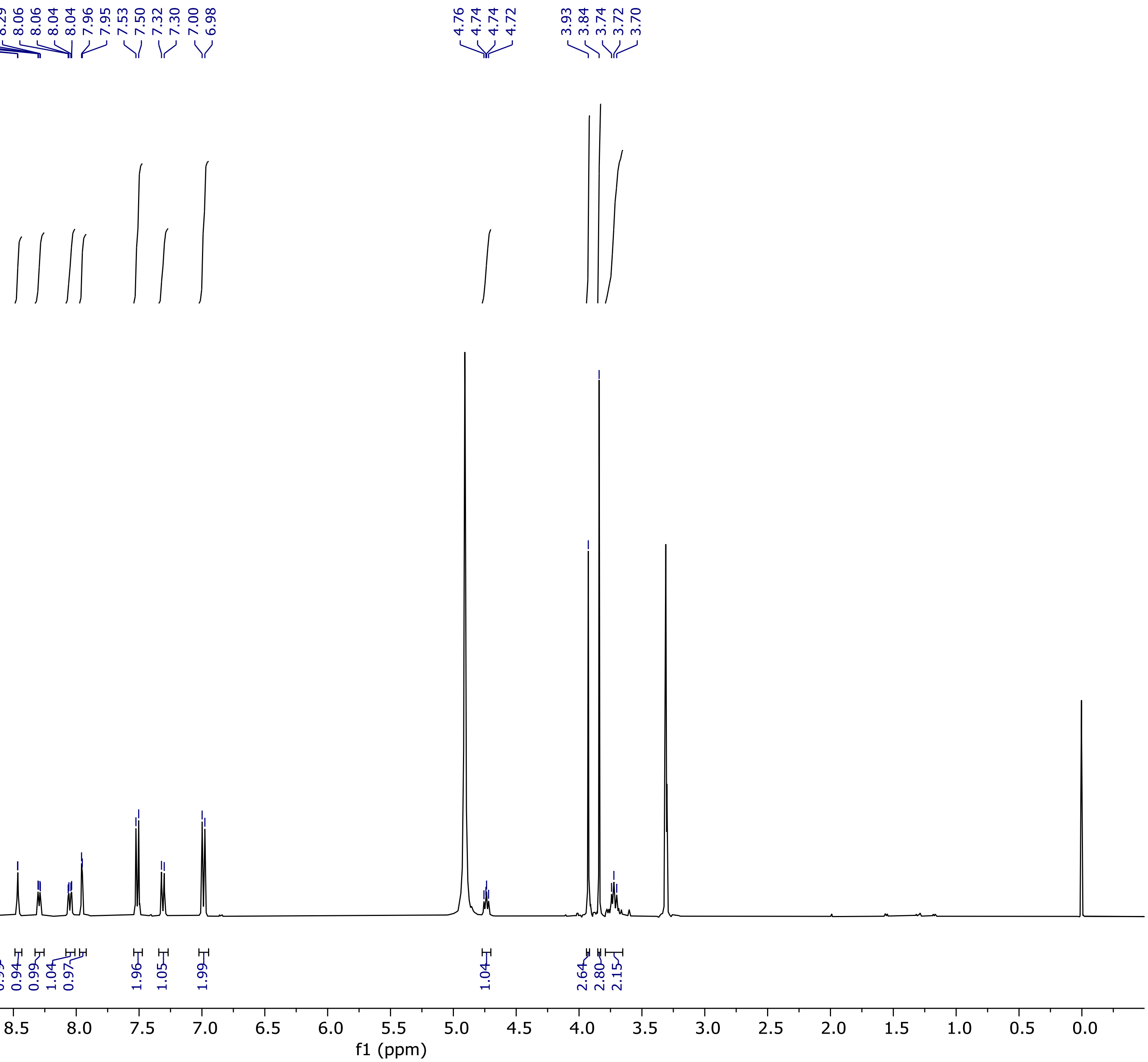

$^{13}C\{^{1}H\}$ NMR (101 MHz, CD_3OD)

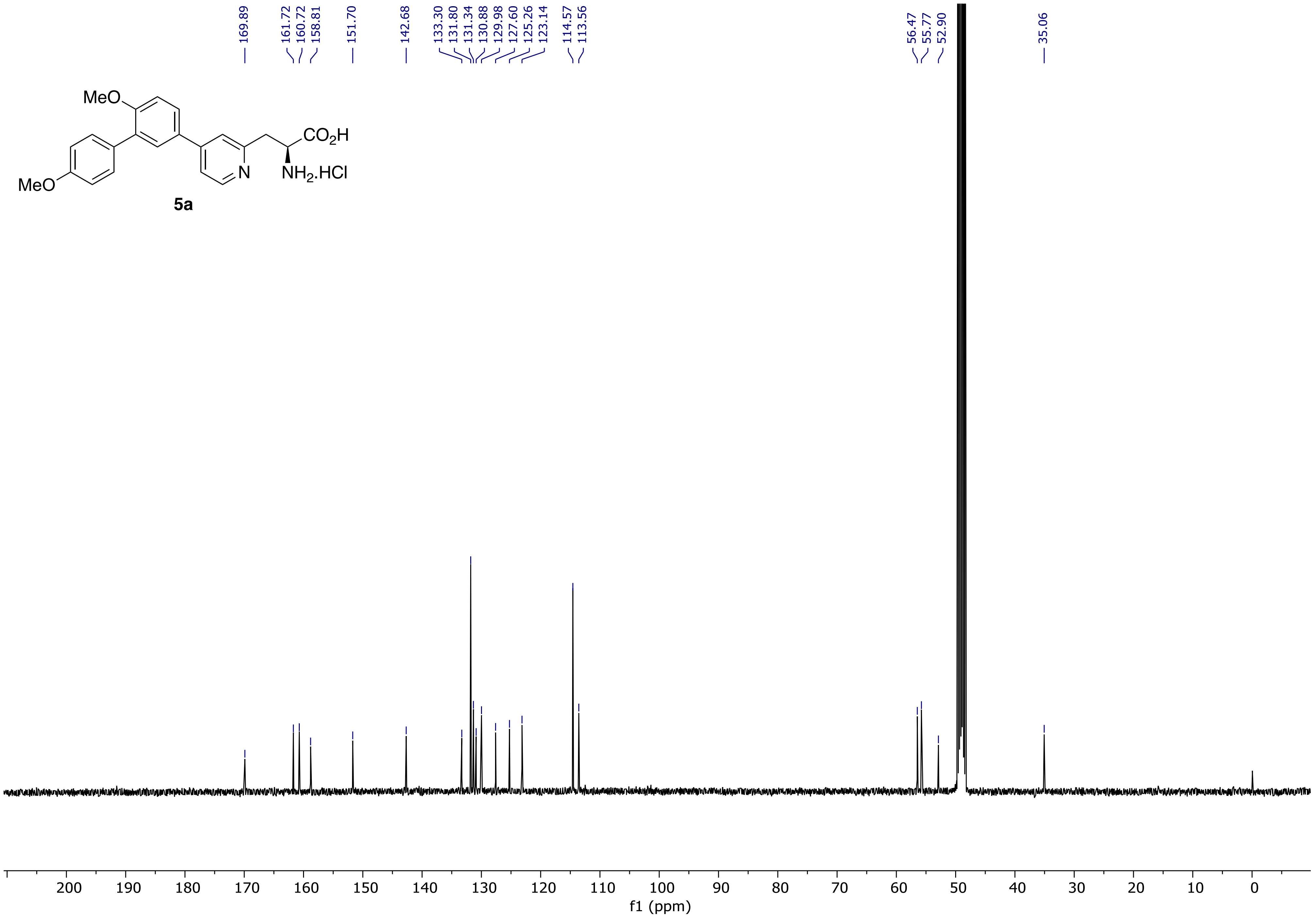

^{1}H NMR (400 MHz, CD_3OD)

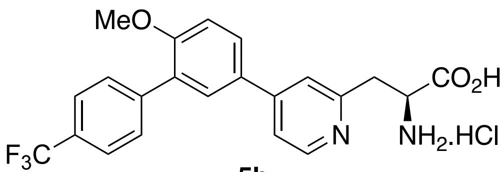

5b

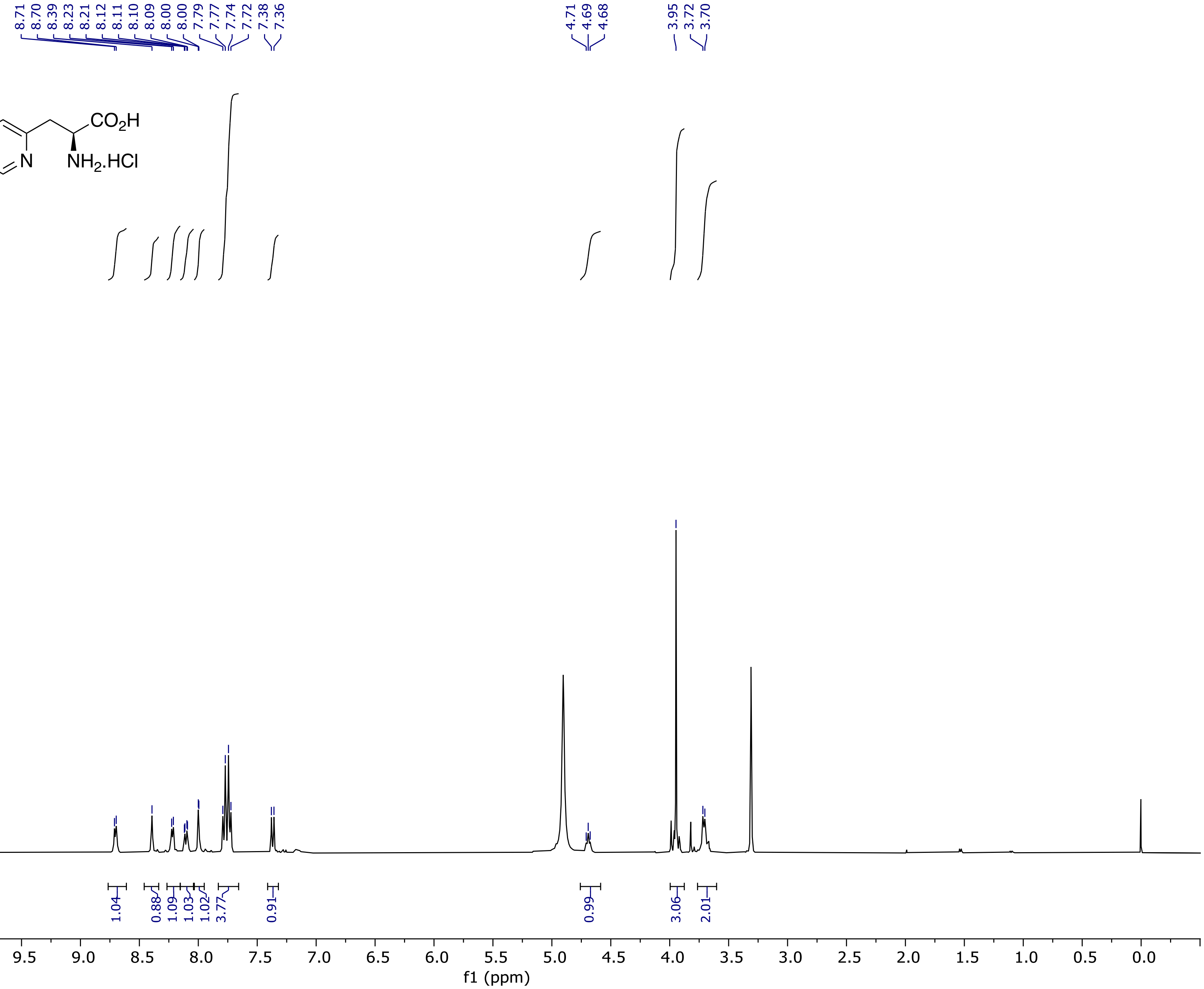

$^{13}C\{^{1}H\}$ NMR (101 MHz, CD_3OD)

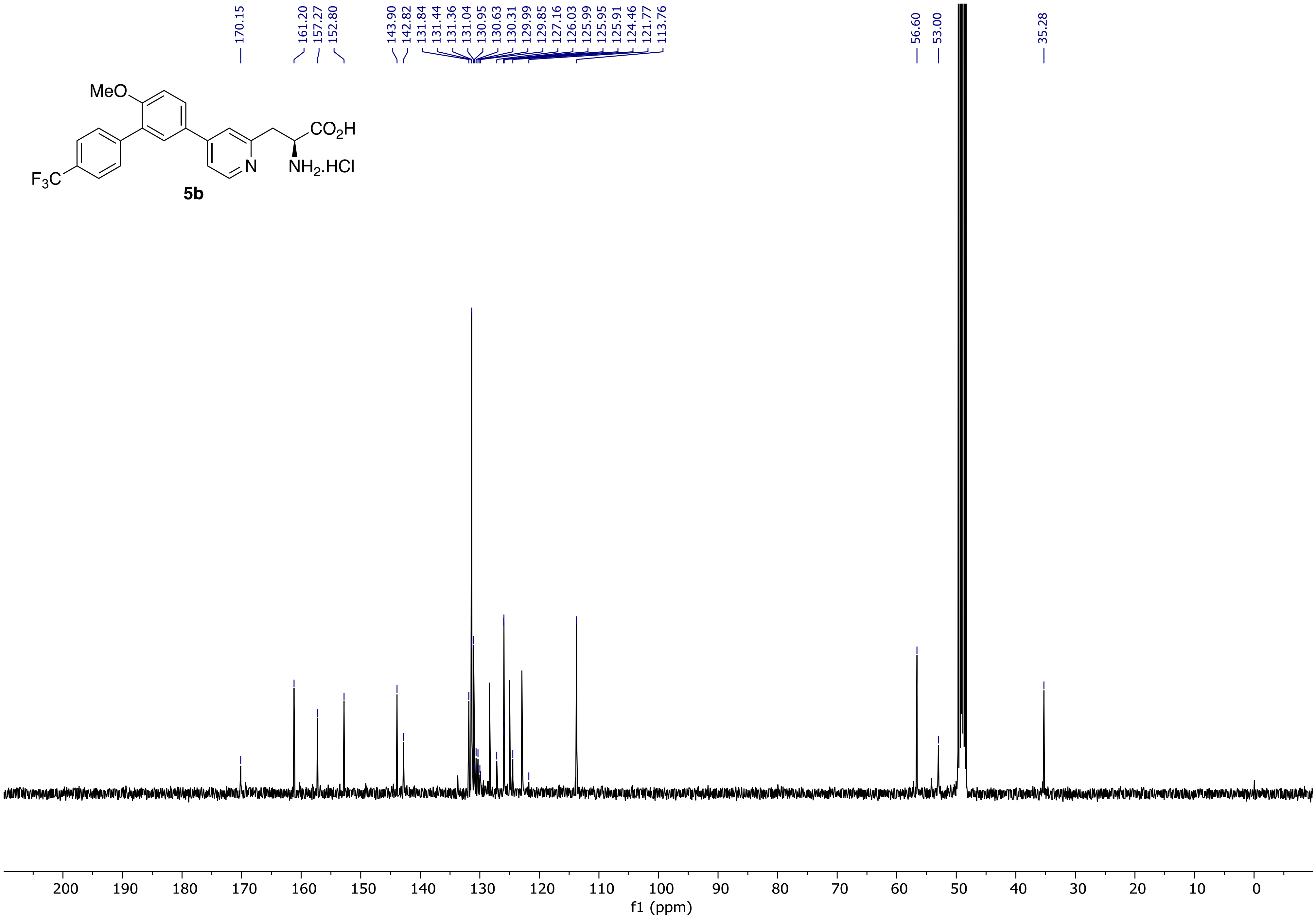

^{1}H NMR (400 MHz, CD_3OD)

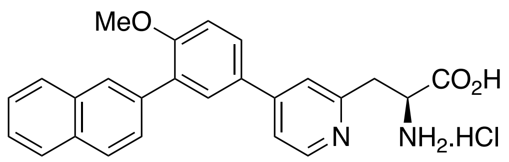

5c

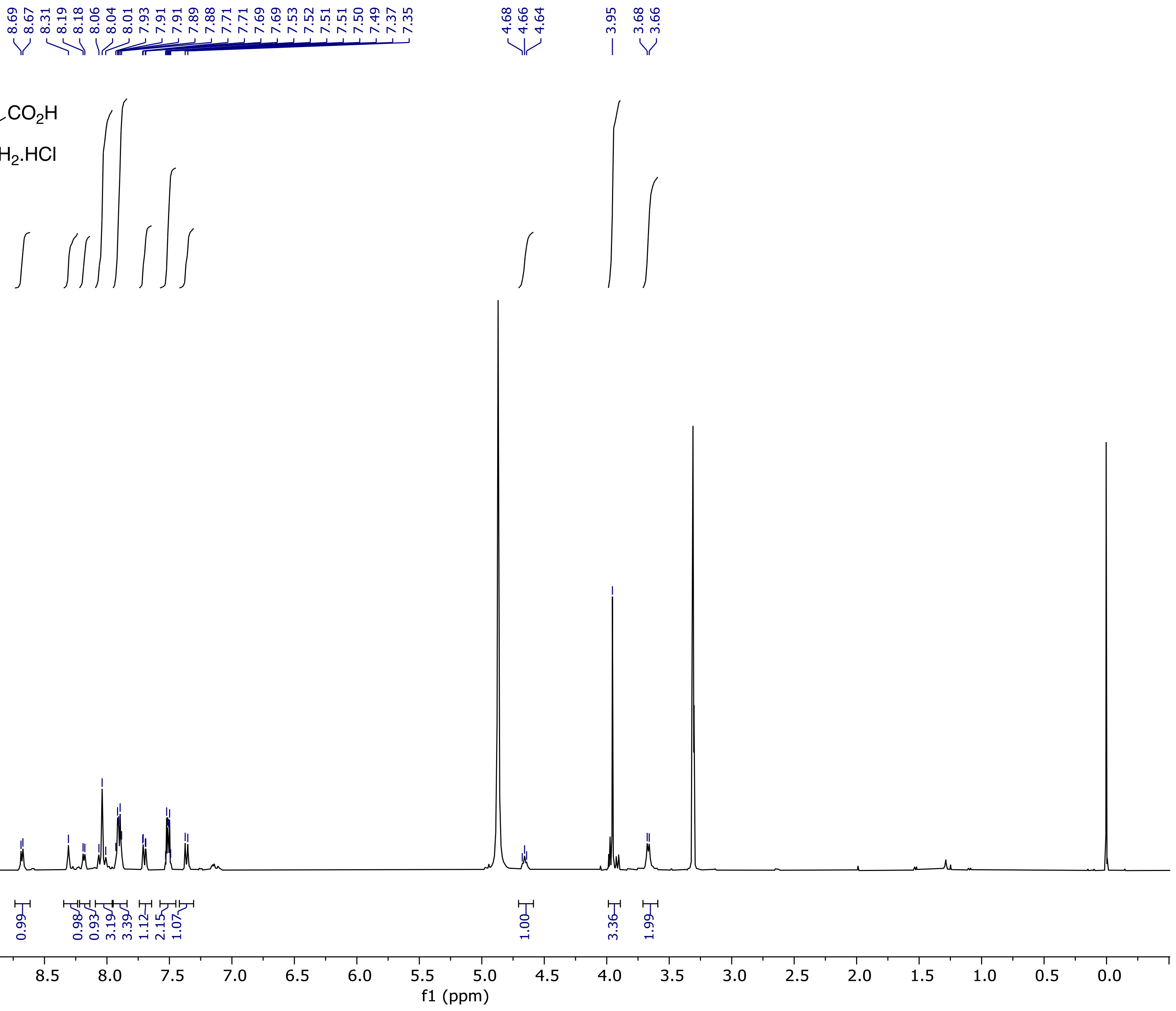

$^{13}C\{^1H\}$ NMR (101 MHz, CD_3OD)

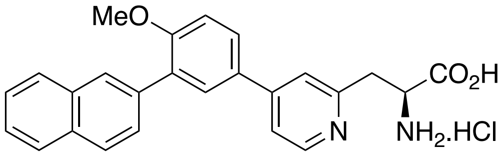

5c

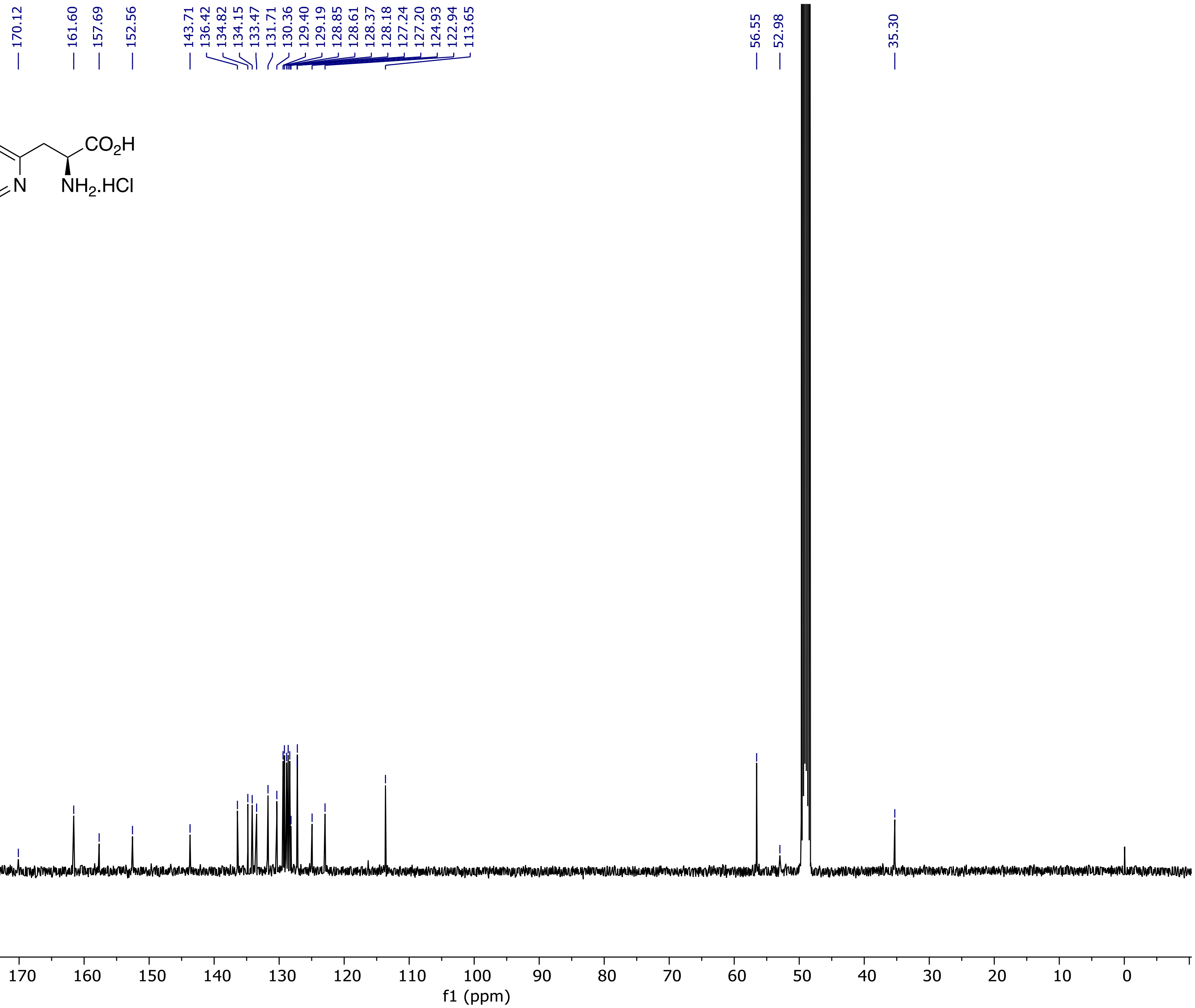

Supplement: Supplementary file 1 — ol4c01951_si_001.pdf [file ol4c01951_si_001.pdf]
